# Critical and Supercritical Spatiotemporal Calcium Dynamics in Beta Cells

**DOI:** 10.3389/fphys.2017.01106

**Published:** 2017-12-22

**Authors:** Marko Gosak, Andraž Stožer, Rene Markovič, Jurij Dolenšek, Matjaž Perc, Marjan S. Rupnik, Marko Marhl

**Affiliations:** ^1^Faculty of Medicine, Institute of Physiology, University of Maribor, Maribor, Slovenia; ^2^Faculty of Natural Sciences and Mathematics, University of Maribor, Maribor, Slovenia; ^3^Faculty of Education, University of Maribor, Maribor, Slovenia; ^4^Faculty of Energy Technology, University of Maribor, Krško, Slovenia; ^5^Center for Applied Mathematics and Theoretical Physics, University of Maribor, Maribor, Slovenia; ^6^Complexity Science Hub, Vienna, Austria; ^7^Institute of Physiology and Pharmacology, Medical University of Vienna, Vienna, Austria

**Keywords:** beta cells, islets of Langerhans, self-organized criticality, intercellular dynamics, calcium waves, glucose oscillations, computational model, confocal calcium imaging

## Abstract

A coordinated functioning of beta cells within pancreatic islets is mediated by oscillatory membrane depolarization and subsequent changes in cytoplasmic calcium concentration. While gap junctions allow for intraislet information exchange, beta cells within islets form complex syncytia that are intrinsically nonlinear and highly heterogeneous. To study spatiotemporal calcium dynamics within these syncytia, we make use of computational modeling and confocal high-speed functional multicellular imaging. We show that model predictions are in good agreement with experimental data, especially if a high degree of heterogeneity in the intercellular coupling term is assumed. In particular, during the first few minutes after stimulation, the probability distribution of calcium wave sizes is characterized by a power law, thus indicating critical behavior. After this period, the dynamics changes qualitatively such that the number of global intercellular calcium events increases to the point where the behavior becomes supercritical. To better mimic normal *in vivo* conditions, we compare the described behavior during supraphysiological non-oscillatory stimulation with the behavior during exposure to a slightly lower and oscillatory glucose challenge. In the case of this protocol, we observe only critical behavior in both experiment and model. Our results indicate that the loss of oscillatory changes, along with the rise in plasma glucose observed in diabetes, could be associated with a switch to supercritical calcium dynamics and loss of beta cell functionality.

## Introduction

Homeostasis of energy-rich nutrients in blood has to cope with behavioral and environmental extremes, such as ingestion of a large meal or prolonged fasting (Schmitz et al., 2008). The anabolic hormone insulin promotes postprandial storage of nutrients and tightly controls their consumption interprandially, thus playing a crucial homeostatic role, which becomes disrupted in obesity and diabetes (Kahn et al., 2014). Similarly to many other hormones, insulin concentration in blood oscillates, with a diurnal (meal-related) component, an ultradian component (period of 80–180 min), and a so called high-frequency component (period of 5–15 min), the last being evolutionary conserved in different mammals, such as humans and mice (Nunemaker, 2005; Satin et al., 2015). It has been established beyond doubt that the oscillations in blood are due to pulsatile release of insulin from the pancreas. In contrast, many questions remain to be answered with regard to how exactly this pulsatile release is brought about and regulated, since the beta cells which sense glucose (and other nutrients) and secrete insulin are scattered throughout the exocrine part of the gland in the form of small organs called islets of Langerhans, of which there are about a thousand in the mouse and about a million in the human pancreas, and each of which contains from a few to a couple of thousand beta cells, together with other endocrine and mesenchymal cells (Dolenšek et al., 2015).

The stimulus-secretion coupling process in beta cells involves entry of glucose (and other nutrients) into the cell and its metabolism to ATP, which in turn decreases the open probability of ATP-sensitive potassium channels, leading to depolarization of plasma membrane, opening of voltage-sensitive calcium channels, a rise in the cytosolic calcium concentration ([Ca^2+^]_c_), and triggering exocytosis (Ashcroft and Rorsman, 2013). In addition to this canonical triggering pathway, there are probably an additional amplifying calcium-dependent (Henquin, 2011) and even a calcium-independent signaling pathway (Aizawa et al., 1998).

Individual, uncoupled beta cells display oscillations of membrane potential and [Ca^2+^]_c_ with a frequency close to the fastest of the abovementioned, but with a large degree of heterogeneity (Tengholm and Gylfe, 2009; Satin et al., 2015). Within islets of Langerhans, a strong intercellular coupling force in the form of intercellular gap junctions consisting of the connexin 36 protein and possibly other modes of cell-cell communication overcome the heterogeneity of individual beta cell oscillators (Bavamian et al., 2007; Konstantinova et al., 2007; Tengholm and Gylfe, 2009; Benninger et al., 2011; Rutter et al., 2017; Skelin Klemen et al., 2017). In whole islets, metabolism, membrane potential, [Ca^2+^]_c_, and secretion of insulin oscillate at a frequency close to the abovementioned 5–15 min due to coupling, however islets also display so called fast oscillations (period of 1–15 s) in the form of bursts of membrane depolarizations (Dean and Matthews, 1970), with accompanying oscillations in [Ca^2+^]_c_ (Gilon et al., 1992; Dolenšek et al., 2013) and insulin secretion (Bergsten, 1995). This fast oscillatory component is responsive to changes in glucose concentration (see below), synchronized in different cells of an individual islet by means of membrane potential and [Ca^2+^] waves (Dolenšek et al., 2013), but does not seem to be entrained into a common rhythm among different islets *in vivo* (Valdeolmillos et al., 1996). In contrast, the slow oscillations of individual islets are not responsive to changes in glucose concentration, but probably are entrained into a common rhythm *in vivo*, yielding the 5–15 min oscillations observed in blood. It is believed that the 5–15 min oscillations are due to oscillations in metabolism and the 1–15 s oscillations are due to a feedback between calcium and potassium channels (Satin et al., 2015). This view is incorporated into a mathematical model called the dual-oscillator model, which in addition to the mixed pattern of carrying slow metabolic and superimposed fast electrical oscillations is also able to account for *in vitro* observations of only the fast or the slow component in absence of the other (Bertram et al., 2007).

Metabolic differences between individual islets (Nunemaker et al., 2005, 2009) can be overcome by weak coupling via an intrapancreatic neural network (Fendler et al., 2009), by negative feedback from the liver (Pedersen et al., 2005; Dhumpa et al., 2014), or both (Satin et al., 2015). According to the recent metronome model, glucose-responsive fast oscillations of individual islets determine the amplitude or pulse mass of the largely stable 5–15 min insulin oscillations (Satin et al., 2015).

Theoretically, an individual islet can respond to an increase in glucose concentration by recruiting more cells into a functional state, by enhancing the response of active cells, or both. Previous experiments have suggested that within a narrow range of glucose concentrations above the threshold concentration, recruitment rapidly saturates and that beyond that, all beta cells within an islet are active all the time, with synchronous membrane potential and [Ca^2+^]_c_ oscillations that increase in plateau fraction with increasing glucose concentrations (Henquin et al., 1982; Henquin, 1987; Valdeolmillos et al., 1989; Santos et al., 1991; Gilon and Henquin, 1995; Jonkers et al., 1999; Jonkers and Henquin, 2001). In sum, according to this view the pulse mass is more importantly determined by enhancing the responses of individual cells than by recruiting new cells (Jonkers et al., 1999; Jonkers and Henquin, 2001). The main shortcoming of previous studies aimed at quantitating the role of recruitment and enhancement is the fact that clusters of beta cells were used instead of islets to ensure spatial resolution at the level of individual cells, and that when whole islets were used, resolution at the level of individual cells was not achieved. Additionally, beta cells have traditionally been stimulated by elevating glucose to supraphysiological concentrations and in a constant, i.e., non-oscillatory manner, not to concentrations slightly above the threshold and in an oscillatory manner, as is probably the case *in vivo*.

Finally, considering the formidable complexity of the mechanism supporting pulsatile insulin release, the teleological question seems appropriate, as to what evolutionary advantage is conferred by pulsatile insulin release. It has been suggested by modeling that pulsatile insulin secretion may be beneficial for the secretory capacity of beta cells in the sense that it allows the readily releasable pool of insulin granules enough time to refill during the resting periods between periods of activity (Pedersen and Sherman, 2009). Additionally, it has been proposed that pulsatile insulin may be important for physiological autocrine effects in islets and expansion of beta cell mass (Tengholm and Gylfe, 2009). On the other hand, experimental evidence points to a greater efficiency of pulsatile insulin on target tissues and continuous insulin leads to internalization, down-regulation of insulin receptors, and post-receptor signaling defects (Pørksen, 2002; Pørksen et al., 2002; Satin et al., 2015). Importantly, disrupted insulin pulsatility has been observed in prediabetes, type 2 diabetes mellitus (T2DM), and even in normoglycaemic relatives of patients with T2DM (Lang et al., 1981; O'Rahilly et al., 1988; Bingley et al., 1992). In T2DM beta cells are also unable to respond to entrainment by imposed oscillations in glucose, indicating a fundamental loss of entrainability (Hollingdal et al., 2000). Furthermore, it has been demonstrated that beta cell coupling is a target of diabetogenic insults, such as lipotoxicity (Hodson et al., 2013), glucotoxicity (Haefliger et al., 2013), and cytokines (Farnsworth et al., 2016; Johnston et al., 2016). Likewise, islets exposed to such insults and islets of Cx36 knockout mice display disrupted calcium waves and synchronization of oscillations (Benninger et al., 2008, 2014). Finally, in Cx36 knockout mice the *in vivo* observed insulin response and pattern of insulin oscillations are disrupted in a manner resembling typical changes in T2DM (Head et al., 2012).

Even though some mathematical models have addressed the collective behavior of coupled beta cells (Smolen et al., 1993; Zimliki et al., 2004) and the propagation of [Ca^2+^] waves (Nittala et al., 2007; Benninger et al., 2008), the development of multicellular computational models is just recently increasingly gaining attention. For the most part, this has been initiated by the growing evidence showing that cell–cell interactions via gap junctions are a prerequisite for proper hormone secretion (Charollais et al., 2000) and that impaired intercellular interactions disrupt normal oscillatory patterns of insulin secretion in a way similar to what occurs in diabetes (Head et al., 2012). Moreover, recent advances in imaging techniques along with the integration of complex system approaches in islet research revealed a complex functional organization of beta cells (Hodson et al., 2013; Stožer et al., 2013b; Cherubini et al., 2015; Gosak et al., 2015, in press; Markovič et al., 2015) that might be predominantly a consequence of cell-to-cell variability and a heterogeneous nature of intercellular interactions (Goel and Mehta, 2013; Barua and Goel, 2016; Cappon and Pedersen, 2016). Detailed analyses of pancreatic [Ca^2+^] waves have shown that the waves originate from specific yet rather randomly distributed sub-regions with elevated excitability (Benninger et al., 2014) or lower metabolic rates (Westacott et al., 2017), thereby giving emphasis also to the spatial aspect of beta cell heterogeneity and the sub-compartmental organization of islets (Markovič et al., 2015). Furthermore, percolating network models have been utilized with the aim to provide a phenomenological insight into the interplay between the coupling architecture and the beta cell activity in health and disease (Benninger et al., 2008; Hraha et al., 2014a,b; Stamper et al., 2014). Most importantly, by these means the existence of a critical behavior that reflects a phase transition between globally active and inactive states was predicted and confirmed both computationally and experimentally (Hraha et al., 2014b). Such a critical transition was suggested to be a general regulatory mechanism that islets utilize in order to leverage cellular heterogeneity, and is characteristic also for a variety of other complex real-life systems (Trefois et al., 2015).

In this vein, many natural systems were found to operate naturally near a critical point (Bak, 1996; Marković and Gros, 2014). The emergent dynamics in such systems is usually associated with the concept of self-organized criticality (SOC), which embraces a power-law distribution of systems' observables. SOC arises in complex systems that are far from equilibrium as a result of interactions of components and is increasingly gaining attention in the context of organizing principles of biological systems. For example, durations of brief awakenings during sleep exhibit a power-law distribution, indicating a scale-invariant dynamic that is typical for systems undergoing SOC (Lo et al., 2004, 2013; Allegrini et al., 2015). The power-law distribution is also characteristic for other human body dynamics, e.g., human gait and human heartbeat; however, the scale invariant feature characterized by 1/*f* scaling is only a hallmark of SOC. Ivanov et al. (Ivanov et al., 2009) showed that while both gait interstride interval and cardiac interbeat interval time series have comparable 1/*f* scaling, they are governed by different mechanisms and lead to different levels of complexity, characterized by monofractal and multifractal properties, respectively. Very recently, as a further example of SOC in human body, clinical evidence has been provided for self-organization of blood pressure regulation (Fortrat and Gharib, 2016). At the single cell level, hallmarks of SOC have been observed in the spatiotemporal organization of [Ca^2+^] waves in individual cardiac myocytes (Nivala et al., 2012) and oocytes (Lopez et al., 2012). However, the greatest progress in this framework has been done in the field of neuroscience where fingerprints of SOC have been identified at different levels of organization, ranging from interacting arrays of neurons or astrocytes to the entire brain (Jung et al., 1998; Beggs and Plenz, 2003; Plenz and Thiagarajan, 2007; Hesse and Gross, 2014). On different scales of observation, patterns of neuronal electrical activity show a high degree of diversity, characterized by a scale free distribution of event (i.e., neuronal avalanches) sizes. Theoretical and experimental work indicates that this form of activity reflects a critical state between a random and an ordered dynamical regime, which is believed to lead to optimal operational abilities (Beggs and Plenz, 2003; Kinouchi and Copelli, 2006). A balanced interplay between network dynamics and topology as well as an activity-dependent adaptability of neuronal networks were identified as leading neurobiological determinants that ensure a robust critical behavior (Levina et al., 2007; Rubinov et al., 2011; Hutt et al., 2014). However, *in vitro* experiments on cortical assemblies have shown that the neuronal activity does not necessarily fall into a critical regime, but can also show subcritical or supercritical states, e.g., during development (Tetzlaff et al., 2010), which has latter been associated with changes in the neuronal network connectivity patterns (Tetzlaff et al., 2010; Massobrio et al., 2015b). Moreover, an excess of large neuronal avalanches, as is characteristic for supercritical dynamical states, has been associated with pathological activity (Meisel et al., 2012; Massobrio et al., 2015a). It has been speculated that deviations from critical behavior reflect the abnormal synchronized firing of neurons involved in the epileptic process (Lehnertz et al., 2009), thereby substantiating the hypothesis that the normal, healthy brain resides in a critical or even slightly subcritical state (Priesemann et al., 2014; Massobrio et al., 2015a). However, in contrast to inanimate matter, for which the SOC principles are well understood, in living systems these mechanisms are still under debate and are conjectured to be a result of different evolutionary or adaptive processes, with structural disorder, order-parameter feedback, and extended criticality as possible explanations (Lovecchio et al., 2012; Moretti and Muñoz, 2013).

Despite growing evidence that critical-like dynamics and power-law scaling imply an efficient design of biological systems, only a few studies have investigated the presence of these features in other multicellular physiological systems. At least in part this lack can be explained by demanding experimental techniques that are required to precisely and noninvasively assess the function of a large number of cells simultaneously and over long periods of time. Previously, it has been demonstrated that the behavior of islets of Langerhans is not as deterministic as once thought (Dolenšek et al., 2013; Hodson et al., 2013; Stožer et al., 2013a,b; Benninger et al., 2014; Hraha et al., 2014a,b; Markovič et al., 2015; Rutter and Hodson, 2015). Thus, in the present study we examined whether fingerprints of SOC can be found in the spatiotemporal pattern of fast [Ca^2+^]_c_ dynamics in interconnected beta cells from islets of Langerhans. We first constructed a computational model of a network of heterogeneous and heterogeneously coupled beta cells and analyzed the statistical organization of intercellular [Ca^2+^]_c_ wave sizes. We simulated beta cell behavior after applying a constant stimulatory concentration of glucose, as well as under an oscillatory stimulation with a slightly lower average glucose concentration, in order to more closely mimic the physiological conditions with oscillating blood glucose and insulin levels. Finally, we compared the model predictions with experimental data obtained by means of confocal functional multicellular calcium imaging of [Ca^2+^]_c_ changes evoked in beta cells in acute tissue slices subjected to the same stimulation protocols as in simulations.

## Materials and methods

### Single cell model

The dynamics of each beta cell is governed by the mathematical model proposed by Bertram et al. (2007). The model combines mitochondrial metabolism, glycolysis and plasma membrane electrical activity and Ca^2+^ activity in the cytosol and in the endoplasmic reticulum (Pedersen and Sherman, 2009). Interconnecting these three compartments represents a general and widely used theoretical framework that provides a firm description of the complex oscillatory patterns, such as compound bursting patterns, observed in experiments. A detailed description of the model equations along with parameter values is given in the Text S1.

### Simulation of glucose stimulation

To simulate the stimulation with glucose, we increased the glucokinase reaction rate parameter *J*_GK_ (Bertram et al., 2007). In case of constant stimulation, i.e., switch from 6 to 8 mM glucose, the parameter was increased from *J*_GK, L_ = 0.04 μMms^−1^ to *J*_GK, H_ = 0.38 μMms^−1^. When we simulated an oscillatory stimulation protocol with glucose, glucokinase reaction rate was smoothly varied between these two values as follows:

(1) $$\begin{array}{l} J_{\text{GK}}(t)=J_{\text{GK},\text{L}}+\frac{1}{\pi }(J_{\text{GK},\text{H}}-J_{\text{GK},\text{L}})\\ \;(\frac{\pi }{2}-\frac{{\text{Tan}}^{-1}\left(k_{w}\left(\left(\left(t+T_{\text{lag}}\right)-T_{P1}\right)\text{ mod }T_{L}\;-T_{P2}\right)\right)}{2}\\ \;+\frac{{\text{Tan}}^{-1}\left(k_{w}\left(\left(\left(t+T_{\text{lag}}\right)\right)\text{ mod }T_{L}\;-T_{P1}\right)\right)}{2}) \end{array}$$

where *k*_*w*_ is a pulse-smoothing parameter set to 0.0005. The period of the wave is given by *T*_*L*_ = 600, 000 *ms*. Durations of the low and high glucose concentration phases are given with the parameters *T*_*P*1_ and *T*_*P*2_. Both parameters were set to 300, 000 *ms*. Lastly, *T*_*lag*_ is used to adjust the onset of the first stimulation. In this manner an oscillatory stimulation protocol was stimulated with a period of 10 min with 5 min intervals of elevated glucose conditions. The course of the periodic variations in glucokinase reaction rate is shown in Figure S1.

### Heterogeneity of beta cells

The pancreatic beta cells exhibit a high degree of heterogeneity, which manifests itself in cell-to-cell variability in their sizes, membrane capacitance, channel densities and conductances, in the rates in glucose-induced insulin synthesis and release, cellular thresholds for glucose utilization and oxidation, etc. (Pipeleers et al., 1994; Benninger and Piston, 2014). In our model we introduce heterogeneity of beta cells by means of a random distribution of some model parameters. Albeit cell-to-cell variability would result in diversity of various model parameters, in the present study we consider only heterogeneity of some crucial parameters in different parts of the machinery that governs the beta cells behavior. In particular, heterogeneity is introduced in the glyceraldehyde 3-P dehydrogenase (GPDH) reaction rate parameter *k*_*GPDH, i*_, glycolytic flux affecting PDH activity *J*_*GPDHbas, i*_, maximal PFK reaction rate *V*_*max, i*_, the membrane capacitance *C*_*i*_, and in *g*_*k*(*ATP*), *i*_ denoting the conductance of ATP-sensitive K^+^ channels. All these parameters were assumed to follow a normal distribution with a relative standard deviation of 30 % with a cut-off of 60 %.

### Network of beta cells

We model the intercellular coupling between beta cells as random geometric graph. Initially *N*=150 cells are arranged randomly in a unit square with a prescribed minimal possible distance (0.04) to ensure a more homogeneous spatial distribution. Connections among the cells represent intercellular communication by means of electrical coupllig, defined as:

(2) $$I_{\text{cpl},i}=g_{i}\overset{N}{\underset{j\neq i}{\sum }}d_{ij}(V_{j}-V_{i}).$$

This coupling term *I*_*cpl, i*_ is added to the equation describing the dynamics of the membrane potential of the *i*-th cell (see Equation s21) in Text S1). The network structure is stored in the coupling matrix *d*. Its *ij*-th element *d*_*ij*_ is set to 1 if the *i*-th cells is connected with the *j*-th cell, whilst otherwise *d*_*ij*_ = 0. In particular, an identical radius range for all cells was chosen as $r_{n}=\sqrt{\langle k\rangle /\left(N\pi \right)}$, where 〈*k*〉 = 6 signifies the average number of connections per cell. Two cells are then connected if they fall within each other's range.

In Equation (M2) *g*_*i*_ stands for the electrical coupling coefficient. Previous studies have indicated a high degree of heterogeneity in the gap junctional conductances between beta cells that exceeds a normal distribution. To implement this feature into our simulations the values of *g*_*i*_ were distributed in accordance to an exponential distribution as:

(3) $$g_{i}=-\ln\left(\text{rand}\left(0,1\right)\right)\langle g_{i}\rangle ,$$

where 〈*g*_*i*_〉 symbolizes the mean value of the distribution (〈*g*_*i*_〉 = 200 pS) and *rand*(0, 1) is a uniformly distributed random number in the interval (0,1). The resulting network models are quite homogeneous and without unconnected components. The intercellular coupling, on the other hand, is rather heterogeneous (see Figure S2).

### Confocal calcium imaging in pancreatic tissue

The preparation of the pancreatic tissue slices and the experimental protocols to monitor changes in intracellular calcium concentration were described in detail before (Speier and Rupnik, 2003; Dolenšek et al., 2013; Stožer et al., 2013b). Briefly, NMRI mice (age 10–20 weeks, both males and females) were sacrificed in order to have their abdomens exposed. 1.9 % low-melting point agarose was injected through the proximal bile duct into the ductal system of pancreas. After transferring the agarose blocks containing isolated pancreas tissue onto the wet ice, the hardened agarose allowed for cutting the soft pancreas tissue into 140 μm thick slices. Slices were incubated in the dye-loading solution containing 6 μM Oregon Green 488 BAPTA-1 AM (OGB-1, Invitrogen, Eugene, Oregon, USA), 0.03% Pluronic F-127 (w/v), and 0.12% dimethylsulphoxide (DMSO, v/v) at room temperature. Recordings of the calcium concentration changes were performed on a Leica TCS SP5 AOBS Tandem II upright confocal system using a 20x Leica HCX APO L water immersion objective (NA 1.0). The excitation wavelength was set to 488 nm and the emission collected in the range 500–650 nm. The sampling rate was 10 Hz. Regions of interest were selected based on cell morphology and exported as time series for off-line analysis. This study was carried out in accordance with the national recommendations. The protocol was approved by the Slovenian Ministry of Agriculture, Forestry and Food U34401-30/2016/U94-01.

### Processing of the recorded time series

**Figure 1:**
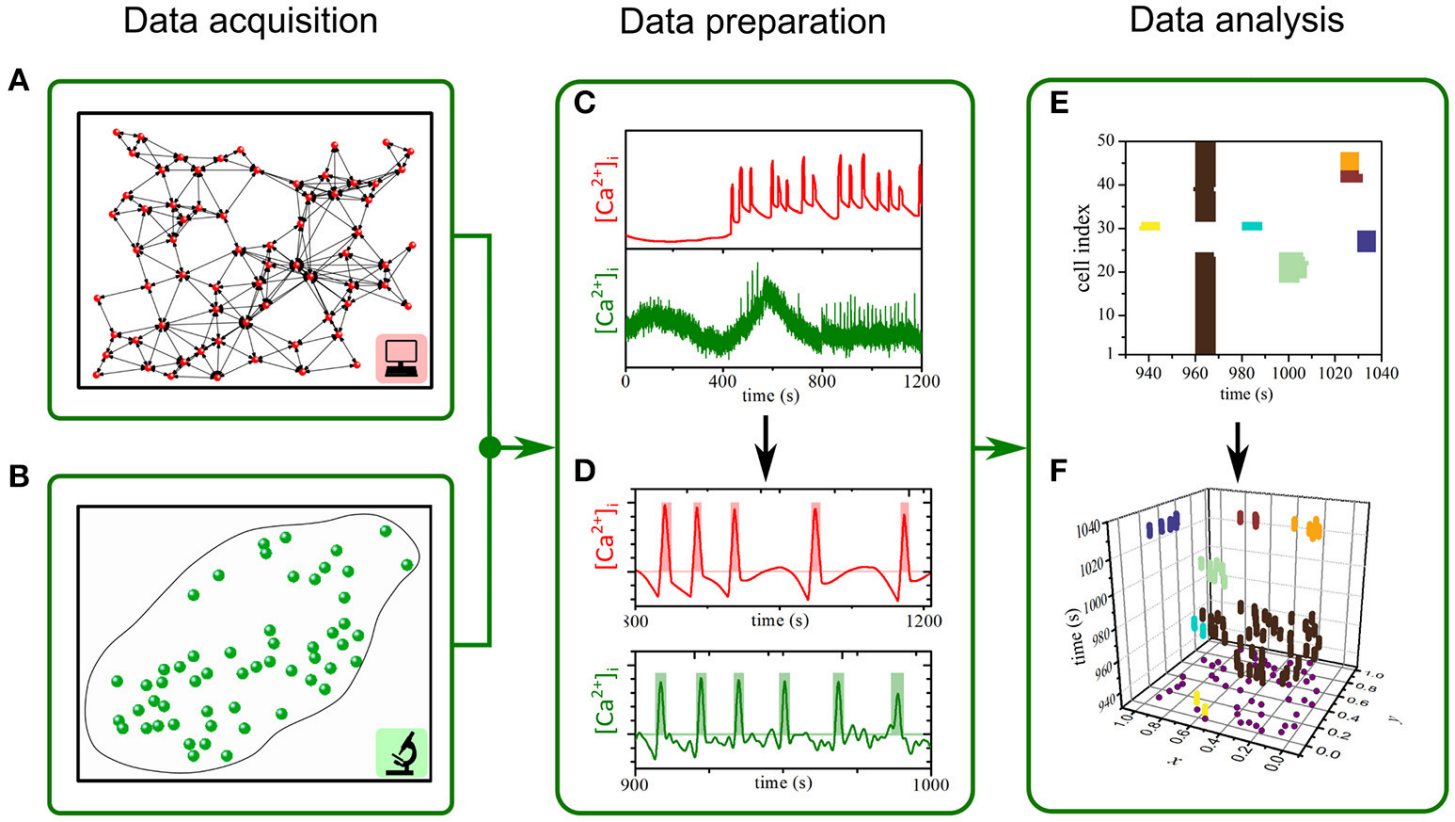
The procedure for assessing spatiotemporal [Ca^2+^]_c_ dynamics in beta cells. **(A,B)**: We used computational simulation (**A**, red) and functional multicellular calcium imaging (**B**, green) to determine beta cell spatial coordinates within networks of beta cells and the individual [Ca^2+^]_c_ activity either in experiments or in simulations **(C,D)**. Oscillations were binarized **(D)** and further processed to extract individual spatio-temporal clusters of [Ca^2+^]_c_ activity **(E,F)**.

Experimentally and numerically obtained [Ca^2+^] traces were initially leveled and smoothed to ensure a consistent and accurate binarization procedure in a two-step signal processing protocol. To extract the fast component of [Ca^2+^] oscillations and to exclude artifacts (i.e., photobleaching) a Butterworth band pass filter (Yao et al., 2012) has been applied to the data sets. The order of the filter has been set to 2 to achieve a steeper frequency cut-off and not to destabilize the filter (resonance disaster). The upper *F*_HIGH_ and lower *F*_LOW_ cut-off frequencies have been determined by visually inspecting all the filtered time traces of a given sample. What followed was the smoothing of the time traces, by means of a sliding window algorithm (Yaroslavsky et al., 2001). The temporal width of the sliding window Δ*t*_*SW*_ has been adjusted to the sampling rate in order to avoid over smoothing of the data (Δ*t*_SW_ = ±2 frames). The solely prepared data has then been used for the binarization of individual beta cell Ca^2+^ activity. The onset and ending of an activation pulse have been determined by computing the first time derivate of individual time traces $\left(\dot{Ca_{i}^{2+}}\right)$ and the corresponding standard deviations of the time traces std $\left(Ca_{i}^{2+}\right)$ as well as time derivates std $\left(\dot{Ca_{i}^{2+}}\right)$. The combined three information's were then used for the binarization. Whenever a local maxima in $\left(\dot{Ca_{i}^{2+}}\right)\left(t\right)$ is >1.5$*\text{std }\left(Ca_{i}^{2+}\right)$ and the corresponding local maxima in $Ca_{i}^{2+}\left(t\right)$ is >1.5$*\text{std }\left(Ca_{i}^{2+}\right)$, we treat the time *t* as the onset of the *n*-th activation time *t*_*START, i*_(*n*). Between the *n*-th local maxima *t*_*START, i*_(*n*) and its first successor *t*_*START, i*_(*n*+1), we search for local minima in $\left(\dot{Ca_{i}^{2+}}\right)$. The discrete time, corresponding to the minima is then set as the end time of the *n*-th activation pulse *t*_*END, i*_(*n*). Values of the binary time trace matrix $Ca_{BIN,i}^{2+}\;$ which lie within the interval *t*_*START, i*_(*n*) and *t*_*END, i*_(*n*) are set to 1 and other values are set to 0. The process is schematically shown in Figures 1C,D.

### Space-time cluster analysis

In order to identify clusters of active cells we implemented the space-time cluster (STC) analysis, similar to the one proposed by Jung (Jung, 1997; Jung et al., 1998). Individual time frames were stacked together to obtain a large space-time cube. Each frame embeds the systems spatial organization, i.e., positions of cells, and the corresponding binary states of the included cells (active or inactive). The time interval between two frames is given by Δ*t*_F_. We start the algorithm by creating a cube around every active cell with a spatial side length and a temporal side length Δ*t*_TSL_. The spatial side length Δ*s* for a given dataset was computed as the average distance between the 6 nearest cells. Typical values of Δ*s* for experimental data were between 20 μm and 25 μm and for the computational model around 0.11. The temporal side length Δ*t*_TSL_ was set to 0.5 s. A STC was then defined as a group of cells, for which the created cubes overlap in a time forward direction. In other words, a cluster results for an isolated intercellular [Ca^2+^] wave that propagates between nearby cells, and its size *p* reflects the number of involved cells and the durations of their [Ca^2+^] pulses in this given event. If two such clusters, i.e., [Ca^2+^] waves, collide, the incoming cluster is joined to the cluster it collides with. The whole procedure from time series preparation to space-time cluster analysis is schematically presented in Figure 1. It should be noted that the size *p* of an individual wave (indicated by the color) actually reflects the volume of a given space-time cluster.

To characterize the spatiotemporal characteristics of intercellular [Ca^2+^] waves, we measured the distribution of cluster sizes. More specifically, we divided the range of sizes' values in a series of non-overlapping intervals with size *p* and counted how many values *N*(*p*) fell into each interval. For both simulated and measured dynamics, we calculated the distribution separately for the activation and for the plateau phase in case of constant stimulation, and over the whole interval (simulated or measured) in case of periodic stimulation. For the representation of the computational results we pooled the data from three (constant stimulation) and four (periodic stimulation) independent simulations runs and for the experimental results we merged the data from three (constant stimulation) and four (periodic stimulation) different tissue slices originating from three different mice. In this manner, the number *N*(*p*) reflects the average number of detected waves with size *p* out of three/four settings. Since the number of cells in the pancreatic slices in different experimental recordings was different, we normalized all slices with respect to the largest detected cluster, i.e., the largest cluster has a size *p* = 1. Finally, the data (experimental and computational) were fitted with a power-law function to qualitatively evaluate if the activity patterns can be treated as critical or supercritical.

## Results

Previous experimental investigations and modeling endeavors have shown both non-trivial temporal activity patterns of beta cell populations and a very complex spatiotemporal organization at the multicellular level (Benninger and Piston, 2014). More specifically, despite gap junctional coupling that fosters the intrinsically heterogenoeus cells to operate in synchrony, a very heterogeneous activity was observed especially in the activation phase when the cells respond to stimulation (Stožer et al., 2013a). Moreover, recent studies also indicate that the beta cell syncytium is functionally organized in a quite complex manner (Stožer et al., 2013b; Markovič et al., 2015; Rutter and Hodson, 2015; Cappon and Pedersen, 2016; Johnston et al., 2016), which provides a basis for non-trivial activity patterns that are less synchronized than once thought. With the aim to explore the spatiotemporal behavior of beta cells, we first built a computational model of interconnected beta cells. The dynamics of individual beta cells is driven by the comprehensive mathematical model proposed by Bertram et al. (2007) that combines glycolysis, and mitochondrial metabolism with plasma membrane electrical activity and [Ca^2+^]_i_ activity. In accordance with previous findings that suggested an extensive heterogeneity among beta cells (Pérez-Armendariz et al., 1991), we introduced variability of some model parameters that govern the beta cell behavior. The cell-to-cell electrical coupling between beta cells was modeleld by means of interactions formed by the random geometric network model, whereby the electrical coupling was assumed to be highly heterogeneous (see Materials and Methods for details). Namely, previous reports and our own observations have shown a high degree of heterogeneity in the gap junctional conductances between beta cells, which do not follow a normal distribution and the values span over a broad interval with a mean value around 200 nS (Pérez-Armendariz et al., 1991). In the continuation, we compare the simulated behavior of a network of interconnected beta cells with experimentally monitored beta cell activity assessed by means of confocal functional multicellular calcium imaging (fMCI).

As already mentioned above, in both simulations and experiments we used two different stimulation protocols: a constant and an oscillatory stimulation. In both cases a typical response to stimulation consisted of a delayed elevation of [Ca^2+^]_i_ with superimposed oscillatory changes of [Ca^2+^]_i_. These oscillations were detected in the form of coordinated [Ca^2+^]_i_ waves spreading across cells. [Ca^2+^]_i_ traces either simulations or experiments were then processed and binarized in order to provide ground for characterization of [Ca^2+^]_i_ signal propagation. To this purpose, we detected and labeled individual intercellular [Ca^2+^]_i_ waves by means of space-time cluster analysis. The whole procedure is schematically presented in Figure 1. Finally, we quantified the intercellular [Ca^2+^]_i_ activity by measuring the spatiotemporal size of waves and looked for the distribution of the number of [Ca^2+^]_i_ waves of a given size [*N*(*p*)] as a function of size (*p*).

### Constant stimulation: computational results

**Figure 2:**
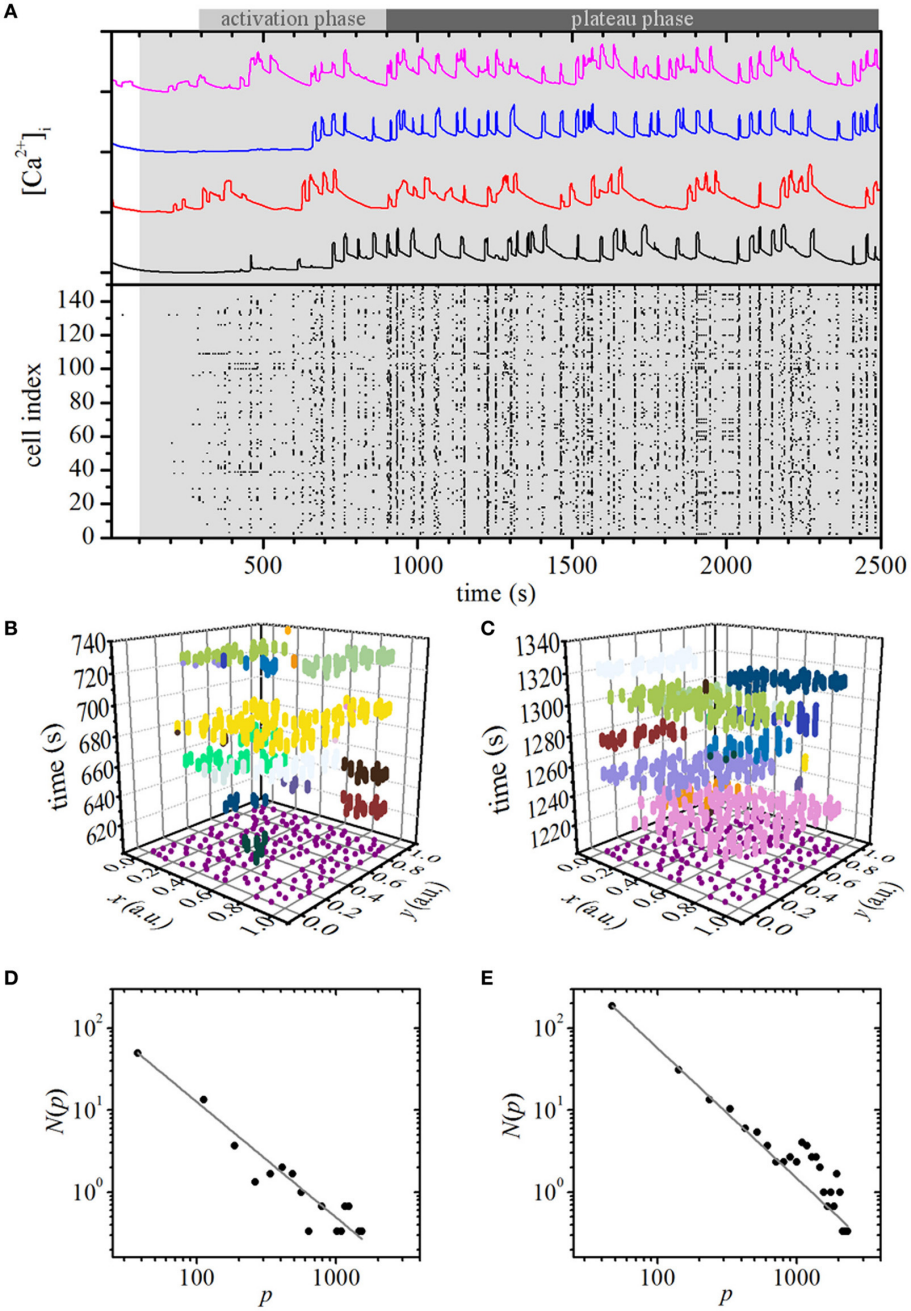
Simulation of constant stimulation: spatiotemporal organization of intercellular [Ca^2+^]_i_ waves in islets. **(A)** Typical computed [Ca^2+^]_c_ responses of four different beta cells after switching to stimulatory conditions (upper panel, gray area indicates stimulatory conditions) and binarization of the computed oscillations of all cells (lower panel). Extracted individual waves as denoted by different colors in space-time graphs during the activation phase **(B)** and in the plateau phase **(C)**. The purple dots in the x-y plane denote the coordinates of individual cells. [Ca^2+^]_i_ wave size distribution of the computed data during the activation **(D)** and plateau phase **(E)**. The gray line indicates a power-law fit with slopes −1.41 and −1.58 for the activation and plateau phase, respectively.

Stimulation with glucose was modeled by simultaneously increasing the glucokinase rate parameter *J*_GK_ from 0.04 to 0.38 at 100 s after the beginning of simulation in all cells in the network. Stimulation initiated an activation phase with a characteristic progressive recruitment of active beta cells with delays spanning 2–10 min, which is in accordance with previous experimental findings (Stožer et al., 2013a). In this stage, the spatiotemporal [Ca^2+^]_i_ activity encompassed smaller clusters of active cells. The activation phase was followed by a plateau phase with characteristic [Ca^2+^]_i_ waves that often encompassed the majority of cells, and, in contrast to the activation phase, displayed a temporally quite ordered and deterministic-like sequence. These observations are illustrated in Figure 2A, whereas a more precise insight into the temporal evolution of the spatiotemporal behavior of the model after stimulation can be observed in Video S1. Next, we quantitatively characterized the [Ca^2+^]_i_ waves, separately for the activation and the plateau phase. To this purpose we first extracted and labeled individual waves, as presented in Figures 2B,C. We then calculated relative wave sizes *p* during the activation (Figure 2D) and the plateau phase (Figure 2E). To ensure a better statistical accuracy, we calculated the distributions as the average of three independent simulation runs. The distribution *N*(*p*) was found to obey a power law in the activation phase, whereas an excess of larger waves was detected in the plateau phase. In sum, this indicates that the persistent stimulation initiates a critical state during the activation phase, which then switches to a supercritical state during the plateau phase.

### Oscillatory stimulation: computational results

**Figure 3:**
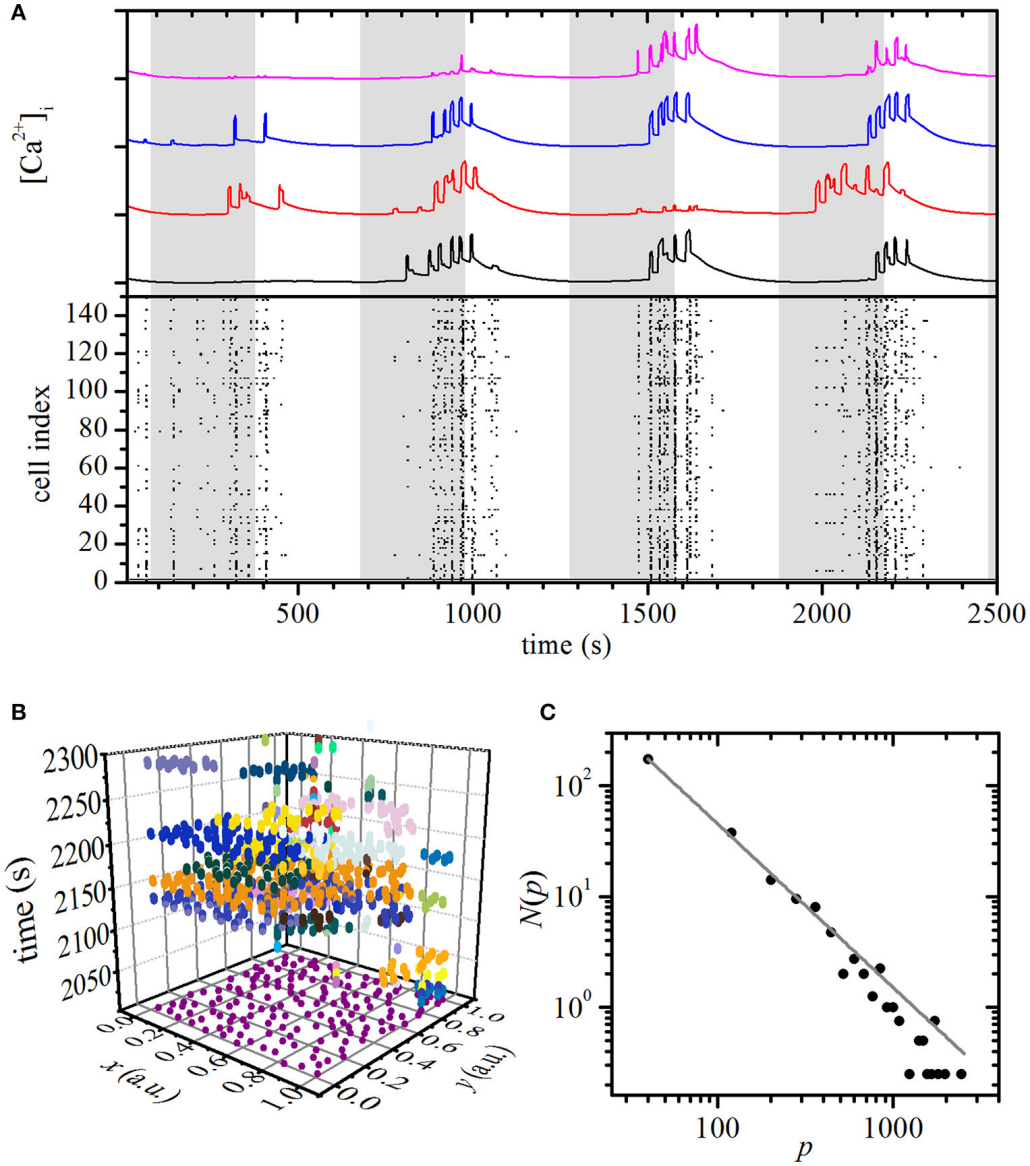
Simulation of oscillatory stimulation: spatiotemporal organization of intercellular [Ca^2+^]_c_ waves in islets. **(A)** Computed [Ca^2+^]_c_ responses of four typical cells during oscillatory glucose stimulation (upper panel) and binarized dynamics of all cells in the network (lower panel). The gray areas denote the stimulatory pulses realized by periodic increases of glucokinase reaction rates. **(B)** Space-time clusters of [Ca^2+^]_i_ activity during the four 5-min glucose stimulations, the colors denote different [Ca^2+^]_i_ waves. **(C)** The distributions *N*(*p*) of spatiotemporal [Ca^2+^]_i_ wave sizes *p*. The gray line indicates a power-law fit with a slope of −1.48.

Using the same computational model, we simulated the beta cell activity during an oscillatory stimulation regime by smoothly changing the glucokinase reaction rate with a period of 10 min, as described in Materials and Methods section. By this means we mimicked oscillations of glucose concentration observed in humans and mice *in vivo* (Head et al., 2012; Satin et al., 2015) in a manner that is compatible with physical limitations of our experimental perifusion setup and is also comparable with other recent reports employing more specialized microperifusion chambers (Pedersen et al., 2013; Dhumpa et al., 2015; Sun et al., 2015). The results are shown in Figure 3. The activity of cells displayed a phase shift of a few minutes with regard to stimulation. Except for the first stimulation pulse, the majority of cells were active at least once in each pulse. Notably, even in the low-stimulatory phases between the pulses (white areas in Figure 3A), cellular activity was observed, but was typically limited to [Ca^2+^]_c_ increases in smaller subgroups of cells. A more detailed insight into the spatiotemporal beta cell dynamics can be seen in Video S2. To evaluate the observed behavior that appeared to be qualitatively different from the one observed in the plateau phase during a constant stimulation, we quantified the intercellular [Ca^2+^]_i_ activity. Figure 3B depicts [Ca^2+^]_i_ waves during the 4th stimulation pulse and the colors denote individual clusters. The average distribution *N*(*p*) of the wave sizes *p* was calculated on the basis of four independent simulation runs and was found to obey a power law, indicating critical behavior of the system during oscillatory stimulation.

### Constant stimulation: experimental results

**Figure 4:**
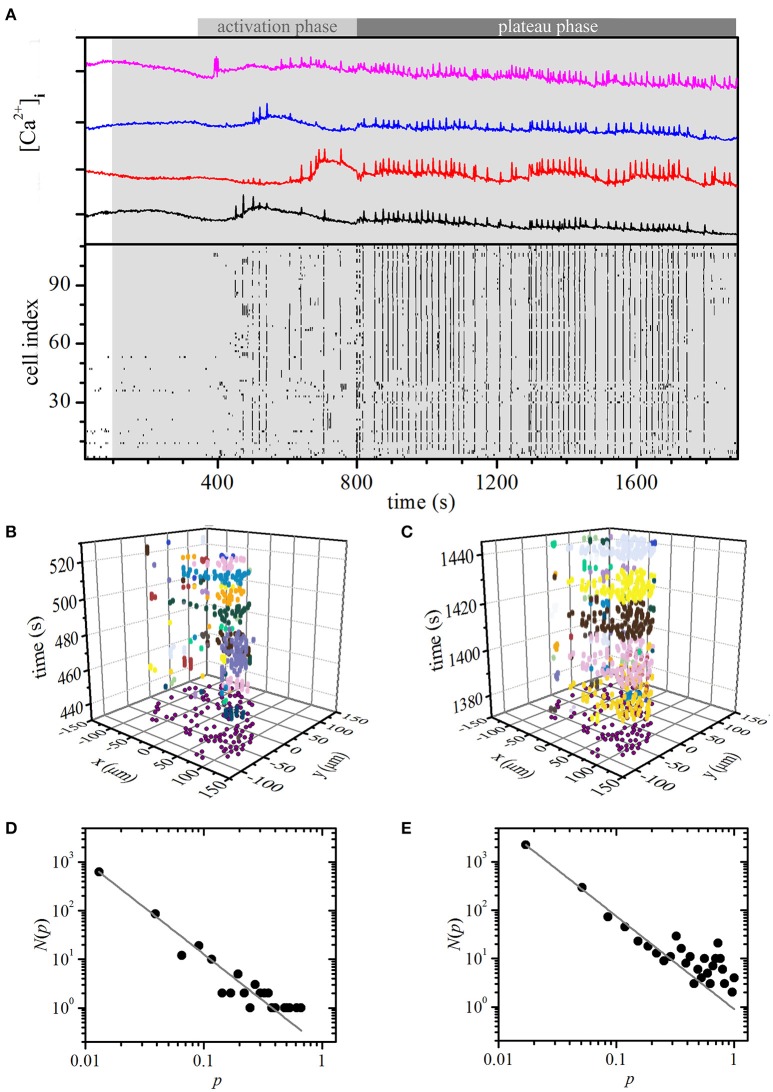
Experimental constant stimulation: spatiotemporal organization of intercellular [Ca^2+^]_c_ waves in islets. **(A)** [Ca^2+^]_i_ responses of four typical cells within an islet to stimulation with 8 mM glucose (upper panel). Binarized oscillations for all cells within the same islet (lower panel). The gray shaded area indicates the switch from 6 to 8 mM glucose. Space-time clusters of [Ca^2+^]_i_ activity in the activation phase (**B**; 300 s < *t* < 800 s) and in the plateau phase (**C**; *t* > 800), the colors denote different [Ca^2+^]_i_ waves. The distributions *N*(*p*) of relative spatiotemporal [Ca^2+^]_i_ wave sizes *p* for the activation phase **(D)** and in the plateau regime **(E)**. The gray line indicates a power-law fit with slopes −1.82 and −1.92 for the activation and plateau phase, respectively.

We stimulated the beta cells within an islet of Langerhans with 8 mM glucose. As in simulations, the cells responded to stimulation with an initial activation phase that was followed by a plateau phase. Characteristic for the activation phase was a transient increase in [Ca^2+^]_c_ and occurrence of fast oscillations, but beta cells were being recruited only gradually during this phase (Figure 4A, 300 s < *t* < 800 s). During the plateau phase that followed the activation phase, all cells were active and displayed repeated and more regular oscillations (Figure 4A, *t* > 800 s). Video S3 shows the temporal evolution of the measured and binarized [Ca^2+^]_c_ activity from the onset of stimulation.

Next, we separately determined [Ca^2+^]_c_ waves during the activation and the plateau phase. Figures 4B,C show such waves during a part of the activation and the plateau phase, respectively, individual waves and their respective sizes are denoted with different colors. Distribution *N*(*p*) of relative wave sizes *p* was determined and plotted on a log-log scale for both the activation (Figure 4D) and the plateau phase (Figure 4E). For the former, *N*(*p*) follows a linear relationship on the log-log scale, whereas for the latter a larger proportion of larger and more global waves could be detected. The distribution signifies the average of three different slices subjected to the same protocol. It should be noted that all of them showed a conceptually very similar behavior, i.e., an activation phase that was followed by a plateau phase with dominating global events, even though the average firing rate and durations of oscillations might be different in different islets. Since the number of cells in each slice was different (111, 125, and 98), we normalized the [Ca^2+^]_c_ wave sizes with respect to the largest one detected in a given slice. Apparently, under constant stimulation, the nature of the spatiotemporal organization changed from a critical regime in the activation phase to a supercritical regime in the plateau phase. These results overlap well with model predictions and, most importantly, substantiate our choice of the model particularities, i.e., a high degree of heterogeneity in properties of individual cells as well as in intercellular coupling.

### Oscillatory stimulation: experimental results

**Figure 5:**
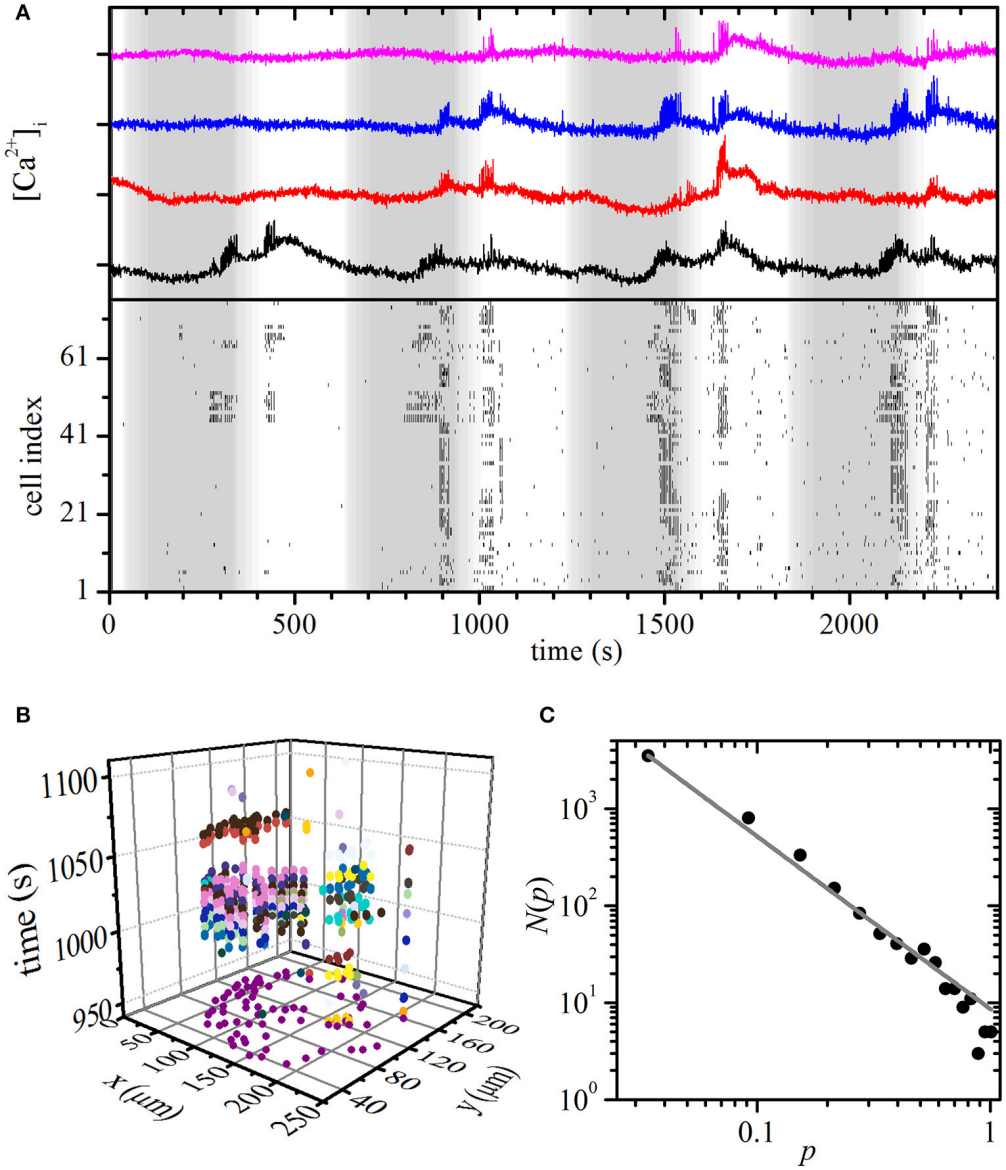
Experimental oscillatory stimulation: spatiotemporal organization of intercellular [Ca^2+^]_i_ waves in islets. **(A)** Typical [Ca^2+^]_i_ responses in four typical cells to an oscillatory stimulation with 8 mM glucose (upper panel). Oscillations are binarized and depicted for all cells within the same islet (lower panel). The gray and white areas denote glucose concentrations of 6 and 8 mM, respectively. **(B)** Space-time clusters of [Ca^2+^]_i_ activity during the second 5 min glucose stimulations, the colors denote different [Ca^2+^]_i_ waves. **(C)** The distributions *N*(*p*) of relative spatiotemporal [Ca^2+^]_i_ wave sizes *p*. The gray line indicates the power-law fit with a slope of −1.78. Note that during glucose nadirs, beta cells remain active.

Finally, we checked if the model correctly predicted the critical spatiotemporal organization of [Ca^2+^]_i_ waves under the oscillatory stimulation protocol. To this purpose, we varied the glucose concentration periodically from 6 to 8 mM with a period of 10 min, whereby each concentration was applied for 5 min. The period of the resulting oscillations lies at the upper end and the amplitude is roughly an order of magnitude larger than what has been described *in vivo*. However, shorter-period and smaller-amplitude oscillatory stimulations are practically impossible to achieve with our perifusion system and this seems to be a challenge also for dedicated microperifusion systems (Roper Industries, US). Results are presented in Figure 5 and Video S4. As in the simulation, a smaller proportion of cells were active already during the first stimulation pulse, however most cells were active during subsequent stimulations. Moreover, different sizes of [Ca^2+^]_i_ waves can be observed and again, a phase shift between glucose stimulation and maximal beta cell activity was observed. We used the same methodological paradigm as with the persistent stimulation to determine [Ca^2+^]_i_ waves. Figure 5B shows spreading of the [Ca^2+^]_i_ waves during the second stimulus, each wave being denoted with a different color. For this stimulatory regime, the distribution *N*(*p*) of the relative sizes *p* of the [Ca^2+^]_i_ waves follows a power law (Figure 5C), as it was predicted by the model. The distribution was calculated on the basis of four different slices subjected to the same protocol and qualitatively identical behavior was observed in all recordings. Since the number of cells in each slice was different (33, 68, 76, and 35), we normalized the [Ca^2+^]_i_ wave sizes with respect to the largest one detected in a given slice. Apparently, oscillations in glucose concentration that mimicked *in vivo* conditions, trapped the beta cell population in a critical regime.

## Discussion

Beta cells respond to stimulation by glucose with an oscillatory activity pattern (Farnsworth and Benninger, 2014). Electrical coupling between beta cells provides the necessary but probably not the only substrate for their coordinated activity and regulated hormone release (Rutter and Hodson, 2013; Benninger and Piston, 2014; Skelin Klemen et al., 2017). However, beta cells within an islet are not completely synchronized. First, measuring a number of different parameters of beta cell function, it has been shown that not all beta cells within an islet respond to stimulation simultaneously, but are progressively recruited into an active state (Schuit et al., 1988; Hiriart and Ramirez-Medeles, 1991; Kiekens et al., 1992; Jonkers and Henquin, 2001; Zarkovic, 2004; Stožer et al., 2013a). Second, the membrane potential and [Ca^2+^] changes spread over the islet in a wave-like manner with a finite speed and do not necessarily always encompass all beta cells in a given islet (Benninger et al., 2008; Dolenšek et al., 2013; Stožer et al., 2013a; Cappon and Pedersen, 2016). These two features reflect the intrinsically heterogeneous nature of beta cells (Benninger et al., 2014; Cappon and Pedersen, 2016), which is retained despite intercellular coupling, most probably for the sake of an at least partly selective and gradual regulation of their function.

To get a more detailed insight into the spatiotemporal organization and the subsequent physiological function of this complex multicellular system, we made use of computational modeling approaches in combination with advanced high spatial and temporal resolution confocal imaging. We developed a multicellular computational model of interconnected beta cells that was based on the theoretical framework of Bertram et al. (2007). By incorporating known particularities, such as cell-to-cell variability in glucose metabolism and conductance of ATP-sensitive K^+^ channels, and a high degree of heterogeneity in the intercellular coupling, we obtained a nice agreement of theoretical results with experimental measurements. A high degree of heterogeneity in coupling was necessary to omit a nearly complete synchronization of cells (see Figure S3). Most importantly, our results showed that [Ca^2+^]_c_ responses and spreading of [Ca^2+^]_c_ events between beta cells after stimulation with non-oscillatory glucose are characterized by a two-phased dynamics: the activation phase and the plateau phase. During the activation phase that lasts around 5 min in case of stimulation with 8 mM glucose, beta cells are recruited and the distribution of [Ca^2+^]_c_ event sizes is characterized by a power-law distribution, suggesting critical behavior. After this initial period, the dynamical nature changes qualitatively and becomes more stable and organized. In the plateau regime that follows, a high number of bigger, more global [Ca^2+^]_c_ events are observed, indicating supercritical behavior of [Ca^2+^]_c_ dynamics.

A constant, i.e., a non-oscillatory elevation in plasma glucose, may not reflect physiological stimulation of beta cells, since blood glucose levels in fasting man and other mammals are oscillating with periods of around 5–15 min (Goodner et al., 1977; Lang et al., 1979). Additionally, the average glucose concentration measured *in vivo* in mice (Kjems et al., 2002; Matyšková et al., 2008) lies just slightly above the *in vitro* determined threshold for glucose-induced metabolic, membrane potential, and [Ca^2+^]_c_ changes (Jonkers and Henquin, 2001; Zarkovic, 2004; Stožer et al., 2013a; Skelin Klemen et al., 2017), and stimulation with 8 mM glucose, albeit being low compared with concentrations that are usually used (e.g., 11.1 or 16.7 mM), is probably still supraphysiologically high. Thus, we attempted to more closely mimic physiological conditions and applied in our simulations and experiments a pulsatile stimulation with 5 min glucose pulses, with an average concentration of 7 mM glucose. This way, the total glucose load that the beta cells received and the maximum concentration reached in the perifusion chamber was the same in the two stimulation protocols (i.e., 20 min × 8 mM vs. 4 × 5 min × 8 mM, maximum = 8 mM). Moreover, during constant stimulation, the glucose concentration was just slightly above the average during oscillatory stimulation and we deliberately chose this value to mimic the continuous rise to hyperglycemia.

Noteworthy, although this was by no means the main aim of our study, our oscillatory protocol also addressed entrainability of islets. We wish to point out that switching from a non-stimulatory to a stimulatory concentration differs significantly from switching between two stimulatory concentrations when it comes to studying entrainability of nonlinear oscillators entrainment (Pedersen et al., 2013). In particular, the former scenario switches the system on and off and always results in entrainment, whereas the latter enables real islet entrainment (Pedersen et al., 2013). Since 6 mM glucose is usually regarded as non-stimulatory, at first our oscillatory protocol seems to correspond to the case of switching the system on and off. However, in our case, even during nadirs of glucose concentration, beta cell activity did not cease and it seems that our protocol therefore enables a true assessment of entrainability and our findings further substantiate recent reports that islets are in fact entrainable by stimuli of periods and amplitudes comparable to ours (Pedersen et al., 2013; Dhumpa et al., 2015; Sun et al., 2015).

During oscillatory stimulation, spatiotemporal organization of [Ca^2+^] waves remained in the critical regime without an excess of global [Ca^2+^] events. Noteworthy, the computational approach is, in contrast to experimental measurements, not restricted by technical limitations and can therefore account for very long observation times. The theoretical results have shown that the periodic stimulation does not lengthen the activation phase and delays the onset of supercritical behavior, but keeps the system essentially trapped in the critical regime. In general, the computational and experimental results are in very good agreement for both constant and periodic stimulation protocols. The only discrepancy occurs in the power-law exponents reflecting critical behavior, whose values are in simulations ranging between −1.41 and −1.58, whereas in experiments the values are a bit more negative and span between −1.78 and −1.94. This difference indicates that despite high levels of physiological complexity, the theoretical model probably still does not cover all the details about the intra- and inter-cellular aspects of beta cell signaling. We believe that especially a more detailed description of beta cell heterogeneity, both metabolic and electrophysiological, could provide a further step toward reality, but future experimental and theoretical work will be needed to address this issue. Moreover, in both computational and experimental results the exponents are found to be a bit more negative in the plateau phase than in the activation phase, which most probably reflects a sharper decay of smaller to intermediate events on account of global calcium waves.

Apparently, periodic entraining of the glucokinase reaction rates confines the supply of energy in the form of ATP, which in turn adjusts the rhythmic activity of ATP-dependent ion channels. This provides in combination with the intercellular coupling, which has either a suppressive effect that prevents uncoordinated and spontaneous activations, or a regulatory role in terms of mediating the synchronizing signal across the islet, the necessary substrate for self-organized activity. To be more precise, as a result of heterogeneities, dynamic and confined regions with elevated excitability emerge, from which [Ca^2+^]_c_ waves are triggered, which also goes in hand with previous findings, where the initiation of waves was associated with regions with a higher glucose metabolism (Benninger and Piston, 2014). The outreach of these waves was found to depend on the metabolic state of surrounding cells and on the local intercellular connectivity. On the other hand, if the glucokinase activity and the subsequent supply of metabolic energy is high, as in prolonged stimulatory conditions, all cells become on average more excitable and the level of their cell-to-cell variability decreases. Consequently, the regulatory role of gap junctional coupling fades and hence an activation of a few cells frequently leads to [Ca^2+^] waves that propagate throughout the whole islet, as is characteristic for supercritical behavior. However, due to the interplay between heterogeneities in cell metabolism, in the level of excitability, and in the intercellular coupling, the transition between the inactive and active beta cell network after switching to stimulatory conditions is not abrupt. Instead, the beta cell recruitment is a time dependent process that evokes an emergent spatiotemporal dynamics characterized by power-law scaling. In this vein, the concept of a functionally heterogeneous beta cell population can be regarded as a key determinant of critical behavior in islets.

Trapping beta cells in a self-organized critical state might be of crucial physiological importance. Namely, criticality seems to be crucial for enabling living tissues reasonable handling of energy and providing efficient functioning and optimized responses to external stimuli. For example, in the field of neuroscience it has been hypothesized that a normal, healthy brain resides in a critical state, which provides the fastest and most flexible adaptation to different challenges from the environment (Chialvo, 2010; Hesse and Gross, 2014; Massobrio et al., 2015a). Tomen et al. (2014) have also shown that cortical networks exhibit optimal neuronal information processing at a near-critical state, i.e., in a narrow region in the phase space at the transition from subcritical to supercritical dynamics. Some studies have even put forward the idea that an optimal physiological neural activity does not perfectly reflect the SOC state but can be characterized as a subcritical regime slightly below the SOC without a separation of time scales, exhibiting the so-called ≫*mélange*≪ of avalanches (Priesemann et al., 2013, 2014). Potential advantages of this marginally subcritical regime slightly below the SOC may be a more efficient information processing, and a safety margin from supercriticality, which has been linked to some pathophysiological disorders. Interestingly, very recently it has been shown that also social systems are poised in the proximity of critical points and by tuning the distance to this point facilitates the system to favor either stability or flexibility (Daniels et al., 2017).

Even within nadirs of our oscillatory stimulation protocol, we were unable to detect any convincing evidence of subcriticality. However, this does not exclude the possibility that *in vivo*, subcriticality in the form of localized individual [Ca^2+^]_c_ might be present. Glucose is a signal that rapidly reaches all beta cells also *in vivo* and it is hard to believe that differences in supply of glucose to different parts of islets or differences in sensitivity of beta cells to glucose could be much higher *in vivo* than in our *in situ* preparation and bring about subcritical behavior (Michau et al., 2016). However, in case of incretins which are also able to evoke [Ca^2+^]_c_ changes in beta cells, evidence exists that especially in human islets, not all beta cells respond to GLP-1 equally well (Hodson et al., 2013, 2014). If differences in sensitivity are great enough, one can imagine that a local [Ca^2+^]_c_ response of a highly sensitive cell might remain localized, especially if the momentary glucose concentration is not great enough to support propagation to neighboring cells (Eddlestone et al., 1984). Likewise, *in vivo*, subcritical behavior of spatiotemporal [Ca^2+^]_c_ dynamics could be brought about by signals able to evoke beta cell [Ca^2+^]_c_ responses that stem from local sources and conceivably stimulate some beta cells more than others, for instance ATP, acetylcholine, or glucagon released from parasympathetic nerve endings or alpha cells (Rodriguez-Diaz et al., 2011a,b; Gylfe et al., 2012). These possibilities remain to be investigated experimentally.

We might further hypothesize that islet tissue operates as a so-called driven subcritical system that is switched to SOC by an entrainment with glucose dynamics. It is well established that glucose oscillations are a hallmark of normal glucose tolerance and islet function (Lang et al., 1979, 1981; Mao et al., 1999; Ritzel et al., 2005). Blood glucose concentration oscillates in both monkeys and humans with a period in the range of the slow insulin oscillations and an amplitude of approximately 1-10 % with respect to the average concentration (Goodner et al., 1977; Lang et al., 1979; Mao et al., 1999). These glucose oscillations seem to be strongly correlated with pulsatile secretion of insulin (O'Meara et al., 1993; Mao et al., 1999; Ritzel et al., 2005; Pedersen et al., 2013; Nunemaker and Satin, 2014; Satin et al., 2015). In fact, it was shown that oscillations of insulin secretion can be entrained by imposed small changes in glucose concentration *in vivo* in normal subjects and that this ability is lost in T2DM (Mao et al., 1999; Hollingdal et al., 2000). It was further suggested that oscillatory glucose actually amplifies the mass of insulin secretory pulses that coincide with imposed glucose stimuli and that the intrinsic frequency of insulin oscillations does not change (Ritzel et al., 2005). This seems in contrast with recent findings that slow oscillations in metabolism and [Ca^2+^]_c_ that are believed to underlie insulin pulses (Pedersen et al., 2013; Sun et al., 2015), as well as insulin secretion can indeed be entrained to glucose (Dhumpa et al., 2015). However, all these contradictions might only be a consequence of still unknown underlying mechanisms for self-organization in human body and the relation of these micro-mechanisms with the emergent macro-phenomena. Recently, Lo et al. (2013) have demonstrated that in contrast to the macroscopic homeostatic equilibrium that describes sleep at the circadian time scale of several hours, the sleep micro-architecture at scales from seconds to minutes exhibits a non-equilibrium behavior of SOC type. They argue that the asymmetry in the transitions between quiet states and avalanches is important as the energy can slowly build up during quiet states, i.e., slowly approaching the critical point, and dissipates rapidly when avalanches occur. Nevertheless, methodologies and novel, more holistic approaches to studying such interconnected physiological processes, occurring in a broad range of space and time scales, are only beginning to emerge. The recently emerging fields of network physiology and network medicine show great potential to provide new insights into how global behavior at the organism level can arise out of micro-mechanisms on the cellular and tissue level, along with networked interactions among different organ systems, to generate health or disease (Bashan et al., 2012; Ivanov et al., 2016).

Our results show that a change in glucose stimulus similar to the one during development of T2DM, i.e., exposure of islets to non-oscillatory and slightly elevated glucose concentration, leads to an excess of large events in the spatiotemporal pattern of fast [Ca^2+^]_c_ oscillations that are believed to determine the pulse mass of insulin oscillations, suggesting that a switch to supercriticality might be an important pathophysiological mechanism in T2DM. This finding corresponds with recent investigations in the brain, showing that epilepsy, for example, is characterized by a supercritical behavior of neurons (Meisel et al., 2012; Priesemann et al., 2014), as well as in a number of other tissues and disease states, such as in obesity and irritable bowel syndrome (caused by a transition in microbial composition), asthma and other pulmonary diseases, depression, inflammation, cancer, and cardiovascular events (for review see Trefois et al., 2015).

In general, an improved understanding of such transitions to supercriticality from normal healthy states during onset and progression of diseases could have important applications in health care. From the viewpoint of preventive, the identification and characterization of early warning signals could predict upcoming critical transitions; and from the view point of curative, the understanding of critical transitions might help us develop more effective therapeutic applications.

## Author contributions

MG, AS, RM, JD, MP, MR, and MM designed and performed the research as well as wrote the paper.

### Conflict of interest statement

The authors declare that the research was conducted in the absence of any commercial or financial relationships that could be construed as a potential conflict of interest.

## Supplementary material

Text S1 Mathematical model and parameter values for beta cells.

Figure S1 The course of the simulated oscillatory stimulation protocol. Variations in the glucokinase reaction rate reflect the oscillatory changes in glucose.

Figure S2 Coupling in the multicellular beta cell model. **(A)** A typical structure of the intercellular network of beta cells. **(B)** The corresponding degree distribution. The beta cell network is quite homogeneous with a mean degree around 6. **(C)** The distribution of the electrical coupling coefficient. The coupling strength is rather heterogeneous and follows an exponential distribution with a mean of 200 pS.

Figure S3 Simulated spatio-temporal activity under constant stimulation with homogeneous coupling. Typical computed [Ca^2+^]_*c*_ responses of four different beta cells after switching to stimulatory conditions (upper panel, gray area indicates stimulatory conditions) and binarization of the computed oscillations of all cells (lower panel). The electrical coupling coefficient was distributed normally with mean 200 pS and relative SD of 30%. In this case very synchronized behavior is obtained without progressive and heterogeneous activations of cells, as observed in experiments.

Video S1 Representative animation of computed and binarized spatiotemporal [Ca^2+^]_*i*_ activity under constant stimulation with glucose.

Video S2 Representative animation of computed and binarized spatiotemporal [Ca^2+^]_*i*_ activity under periodic stimulation with glucose.

Video S3 Movie of experimentally measured and binarized [Ca^2+^]_*i*_ activity under constant stimulation with 8 mM glucose from the onset of glucose increase.

Video S4 Movie of experimentally measured and binarized [Ca^2+^]_*i*_ activity under periodic stimulation with 6-8-6-8-6-8-6 mM glucose.
