# Supplementary material for: Critical and Supercritical Spatiotemporal Calcium Dynamics in Beta Cells
Source: Front Physiol. 2017 Dec 22;8:1106. doi: 10.3389/fphys.2017.01106 (PMC5743929; doi:10.3389/fphys.2017.01106)
Supplement: Text S1 — Mathematical model and parameter values for beta cells. [file Presentation1.PDF]

Supplementary Material

Critical and Supercritical Spatiotemporal Calcium Dynamics in Beta Cells

Marko Gosak[1,2], Andraž Stožer[1], Rene Markovič[2,3,4], Jurij Dolenšek[1], Matjaž Perc[2,5,6], Marjan Slak Rupnik[1,7,*], Marko Marhl[2,3,*]

[1] Institute of Physiology, Faculty of Medicine, University of Maribor, Taborska 8, SI-2000 Maribor, Slovenia

[2] Faculty of Natural Sciences and Mathematics, University of Maribor, Koroška cesta 160, SI-2000 Maribor, Slovenia

[3] Faculty of Education, University of Maribor, Koroška cesta 160, SI-2000 Maribor, Slovenia

[4] Faculty of Energy Technology, University of Maribor, Hočevarjev trg 1, SI-8270 Krško, Slovenia

[5] CAMTP – Center for Applied Mathematics and Theoretical Physics, University of Maribor, Mladinska 3, SI-2000 Maribor, Slovenia

[6] Complexity Science Hub, Josefstädterstraße 39, A-1080 Vienna, Austria

[7] Institute of Physiology and Pharmacology, Medical University of Vienna, Schwarzspanierstraße 17A, AU-1090 Vienna, Austria

*** Corresponding Authors**: marjan.slakrupnik@muv.ac.at, marko.marhl@um.si

1 Supplementary Information – Mathematical model and parameter values for beta cells

The computational model consists of N interconnected beta cells. The dynamics of each beta cell is governed by the mathematical model proposed by Bertram *et al.* [1]. Many model parameters and variables are cell specific and are additionally labeled with and subscript i, meaning that it accounts for the i-th cell. The model is capable to generate fast and slow oscillations in cytosolic Ca^{2+} concentration that are typically observed in islets by interconnecting slow oscillations during glycolysis, a mitochondrial component which transforms the glycolytic input to ATP output, and an electrical/calcium component that responds to ATP from the mitochondria by changing the pattern of electrical activity. The glycolytic part of the mathematical model was proposed by Smolen [2] with the aim to mimic the kinetics of skeletal muscle phosphofructokinase (PFK) in regard to AMP, ATP, fructose 6-phosphate concentration (F6P), glucose 6-phosphate (G6P), product fructose 1,6-bisphosphate concentration (FBP). A pivotal feature is the activity level of the enzyme glucokinase

(GK), which is the glucose sensor in beta cells [3], providing the input for the enzyme phosphofructokinase (PFK). The glycolytic part of the model affects the mitochondrial model compartment, which consists of four variables namely NADH, ADP, and Ca^{2+} concentrations and the inner membrane potential. The mathematical model for the mitochondrial part was introduced in Ref. [4]. Since the model dose not explicitly take into account pyruvate in the glycolytic model, J_{GPDH} (which should be proportional to pyruvate concentration), is used as input for the PDH reaction. The mitochondrial model compartment additionally interacts with the electrical/calcium model compartment, which describes the electrical activity of the plasma membrane, the Ca^{2+} concentrations in the cytosol and the endoplasmic reticulum (Ca_{c} and Ca_{ER}), and the ADP concentration in the cytosol ($\mathrm{ADP_c}$). The electrical/calcium was taken from [5] and is used to simulate the temporal evolution of the plasma membrane potential and the cytosolic and endoplasmic reticulum Ca^{2+} concentration.

The glycolictic part of the model is described with the following equations:

$$J_{\mathrm{GPDH},i} = k_{\mathrm{GPDH},i}\sqrt{\frac{FBP_i}{1\mu\mathrm{M}}}\,\mu\mathrm{Mms}^{-1}, \tag{S1}$$

$$J_{\mathrm{PFK},i} = V_{\mathrm{max},i}\frac{(1-\lambda)w_{1110} + \lambda\sum_{abc} w_{abc1}}{\sum_{acbd} w_{abcd}}, \tag{S2}$$

$$\frac{\mathrm{d}FBP_i}{\mathrm{d}t} = J_{\mathrm{PFK},i} - \frac{1}{2}J_{\mathrm{GPDH},i}, \tag{S3}$$

$$\frac{\mathrm{d}G6P_{,i}}{\mathrm{d}t} = J_{\mathrm{GK}} - J_{\mathrm{PFK},i}, \tag{S4}$$

where $J_{\mathrm{GPDH},i}$ stands for the glyceraldehyde 3-P dehydrogenase (GPDH) reaction rate and $J_{\mathrm{PFK},i}$ is the PFK reaction rate. G6P is assumed to be in rapid equilibrium with F6P (F6P = 0.3 G6P). J_{GK} is the glucokinase reaction rate, which is treated as a glucose dependent parameter. The PFK reaction rate is given by:

$$w_{abcd} = \frac{1}{f_{13}^{ac} f_{23}^{bc} f_{41}^{ad} f_{42}^{bd} f_{43}^{cd}}\left(\frac{AMP}{K_1}\right)^a \left(\frac{FBP}{K_2}\right)^b \left(\frac{F6P}{K_3}\right)^c \left(\frac{ATP}{K_4}\right)^d, \tag{S5}$$

where w_{abcd} reassembles the fraction of PFK in state *abcd*, whereby a, b, c and d are either 1 or 0. Parameter values for the glycolytic model are given in Table 1.

Table 1: Parameter values for the glycolictic compartment model.

$\overline{k_{\mathrm{GPDH},i}} = 0.00078$ μMms^{-1}		$\overline{V_{\max,i}} = 5.8$ μM ms^{-1}	λ = 0.06	
$K_1 = 30$ μM	$K_2 = 1$ μM	$K_3 = 50000$ μM	$K_4 = 220$ μM	
f_{13}=0.02	$f_{23} = 0.2$	$f_{41} = 20$	$f_{42} = 20$	$f_{43} = 20$

The PDH reaction in the mitochondrial part of the model is given by:

$$J_{\mathrm{PDH},i} = \left(\frac{p_1 NAD_{\mathrm{m},i}}{p_2 NAD_{\mathrm{m},i} + NADH_{\mathrm{m},i}}\right)\left(\frac{Ca_{\mathrm{m},i}}{p_3 + Ca_{\mathrm{m},i}}\right)\left(J_{\mathrm{GPDH},i} + J_{\mathrm{GPDHbas},i}\right), \qquad \text{(S6)}$$

The parameter values of p_1 - p_3 and all other mitochondrial parameters are given in Table 2 and taken from Bertram et al. [4]. The first factor in Eq. (S6) reflects the positive effect of $NAD_{\mathrm{m},i}$ and the negative effect of $NADH_{\mathrm{m},i}$ on the PDH reaction rate. In addition, the activity level is increased by mitochondrial Ca^{2+}, labeled as $Ca_{\mathrm{m},i}$, as reflected in the second factor. Finally, PDH activity is driven by glycolytic flux, as represented by $J_{\mathrm{GPDH},i}$ and a small basal glycolytic flux level $J_{\mathrm{GPDHbas},i}$. In turn the $NAD_{\mathrm{m},i}$ dynamics is governed by:

$$\frac{\mathrm{d}NADH_{\mathrm{m},i}}{\mathrm{d}t} = \gamma\left(J_{\mathrm{PDH},i} - J_{\mathrm{O},i}\right). \qquad \text{(S7)}$$

In Eq. (S7) γ is used as a conversion factor and $J_{\mathrm{O},i}$ is the oxygen consumption rate during $NADH_{\mathrm{m},i}$ to $NAD_{\mathrm{m},i}$ convertation, which is defined as:

$$J_{\mathrm{O},i} = \left(\frac{p_{4p_{1,i}} NADH_{m,i}}{p_{5p_{1,i}} + NADH_{m,i}}\right)\left(\frac{1}{1 + e^{\frac{\Delta\psi_i - p_{6p_{1,i}}}{p_{7,i}}}}\right), \qquad \text{(S8)}$$

where $\Delta\psi_i$ represent the mitochondrial inner membrane potential. It schould be noted, that the model assumes nucleotide conservation:

$$NAD_{\mathrm{tot}} = NADH_{\mathrm{m},i} + NAD_{\mathrm{m},i}. \qquad \text{(S9)}$$

The dynamics of the mitochondrial inner membrane potential is dictacted by the equation:

$$C_{\mathrm{m}}\frac{\mathrm{d}\Delta\psi_i}{\mathrm{d}t} = J_{\mathrm{H,res},i} - J_{\mathrm{H,leak},i} - J_{\mathrm{H,apt},i} - J_{\mathrm{ANT},i} - J_{\mathrm{NaCa},i} - 2J_{\mathrm{uni},i}, \quad \text{(S10)}$$

where the mitochondrial inner membrane capacitance is given by C_{m}. The flux $J_{\mathrm{H,res},i}$ represents the flux through respiration-driven proton pumps, which are powerd by O_2 consumption and is defined by the expression:

$$J_{\mathrm{H,res},i} = \left(\frac{p_8 NADH_{\mathrm{m},i}}{p_9 + NADH_{\mathrm{m},i}}\right)\left(\frac{1}{1 + e^{\frac{\Delta\psi_i - p_{10}}{p_{11}}}}\right). \quad \text{(S11)}$$

The second flux in Eq. (S10) $J_{\mathrm{H,leak}}$ linearly deepHendent on the membrane potential, coused by proton leakage and is computed as:

$$J_{\mathrm{F1F0},i} = \left(\frac{p_{13}}{p_{13} + ATP_{\mathrm{m},i}}\right)\left(\frac{p_{16}}{1 + e^{\frac{p_{14-\Delta\psi_i}}{p_{15}}}}\right), \quad \text{(S12)}$$

$$J_{\mathrm{H,atp},i} = 3J_{\mathrm{F1F0},i}, \quad \text{(S13)}$$

$$J_{\mathrm{H,leak},i} = p_{17}\Delta\psi_i + p_{18}. \quad \text{(S14)}$$

The producd ATP in the mitochondria is transported out to the cytosol and exchanged with ADP. Flux through the translocator, $J_{\mathrm{ANT},i}$, is given by:

$$J_{\mathrm{ANT},i} = p_{19}\frac{RAT_{\mathrm{m},i}}{RAT_{\mathrm{m},i} + p_{20}}e^{0.5FRT\Delta\psi_i}. \quad \text{(S15)}$$

where $RAT_{\mathrm{m},i} = ATP_{\mathrm{m},i}/ADP_{\mathrm{m},i}$ and $FRT = F/RT = 0.037\ \mathrm{mV}^{-1}$ ¼ is Faraday's constant divided by the gas constant and the temperature.

The rate $J_{\mathrm{uni},i}$ of Calcium ions through the Ca^{2+} uniporters, through which Calcium ions enter the mitochondria through Ca^{2+} is given by:

$$J_{\mathrm{uni},i} = (p_{21}\Delta\psi_i - p_{22})Ca_{\mathrm{c},i}^2 \quad \text{(S16)}$$

where $Ca_{c,i}$ is the cytosolic Ca^{2+} concentration. Calcium transportation from the mitochondria to the cytosol by Na^{+}/Ca^{2+} exchangers is given by the flux $J_{NaCa,i}$:

$$J_{\mathrm{NaCa},i} = p_{23} \frac{Ca_{\mathrm{m},i}}{Ca_{\mathrm{c},i}} e^{p_{24}\Delta\psi_i} \tag{S17}$$

The mitochondrial Ca^{2+} concentration then changes according to:

$$\frac{\mathrm{d}Ca_{\mathrm{m},i}}{\mathrm{d}t} = -f_{\mathrm{m}} J_{\mathrm{m},i}, \tag{S18}$$

where f_{m} symbolizes the fraction between free Ca^{2+} and $J_{m,i} = J_{\mathrm{NaCa},i} - J_{\mathrm{uni},i}$. Lastly, the the mitochondrial dynamics describing the ADP concentration (in mM) is given by:

$$\frac{\mathrm{d}ADP_{\mathrm{m},i}}{\mathrm{d}t} = \gamma\left(J_{\mathrm{ANT},i} - J_{\mathrm{F1F0},i}\right). \tag{S19}$$

The model takes additionally assumes that the total concentration of adenine nucleotides $A_{\mathrm{m,tot}}$ conserved:

$$A_{\mathrm{m,tot}} = ADP_{\mathrm{m},i} + ATP_{\mathrm{m},i}. \tag{S20}$$

Table 2. Parameter values for the mitochondrial compartment

$p_1 = 400$	$p_2 = 1$	$p_3 = 0.01$ μM	$p_4 = 0.6$ μM ms^{-1}
$p_5 = 0.1$ μM	$p_6 = 177$ mV	$p_7 = 5$ mV	$p_8 = 7$ μM ms^{-1}
$p_9 = 0.1$ μM	$p_{10} = 177$ mV	$p_{11} = 5$ mV	$p_{13} = 10$ μM
$p_{14} = 190$ mV	$p_{15} = 8.5$ mV	$p_{16} = 35$ μM ms^{-1}	$p_{17} = 0.002$ μM ms^{-1} mV^{-1}
$p_{18} = -0.03$ μM ms^{-1}	$p_{19} = -0.03$ μM ms^{-1}	$p_{20} = 2$	$p_{21} = 0.04$ μM^{-1} ms^{-1} mV^{-1}
$p_{22} = 1.1$ μM ms^{-1}	$p_{23} = 0.01$ ms^{-1}	$p_{24} = 0.016$ mV^{-1}	$\overline{J_{\mathrm{GPDHbas},i}} = 0.0005$ μM ms^{-1}
$f_{\mathrm{m}} = 0.01$	$NAD_{\mathrm{tot}} = 10$ mM	$A_{\mathrm{m,tot}} = 15$ mM	$C_{\mathrm{m}} = 1.8$ μM mV^{-1}

The relationship between the ionic currents $I_{\mathrm{k},i}$, $I_{\mathrm{Ca},i}$, $I_{\mathrm{k(Ca)},i}$ and $I_{\mathrm{k(ATP)},i}$ and the plasma membrane potential V_i used in the electrical/calcium part of the modes is given by:

$$C_i \frac{\mathrm{d}V_i}{\mathrm{d}t} = -I_{\mathrm{k},i} + I_{\mathrm{Ca},i} + I_{\mathrm{k(Ca)},i} + I_{\mathrm{k(ATP)},i} + g_i \sum_{j \neq i}^{N} d_{ij}(V_j - V_i), \tag{S21}$$

where the ion fluxes $I_{\mathrm{k},i}$, $I_{\mathrm{Ca},i}$, $I_{\mathrm{k(Ca)},i}$ and $I_{\mathrm{k(ATP)},i}$ represent Ca^{2+} current, K^+ current, Ca^{2+} depended K^+ current and ATP-sensitive K^+ current, respectively. The capacitance of the membrane is given by C. The dynamics of the individual ionic currents is governed by the following set of equation:

$$I_{\mathrm{k},i} = \bar{g}_{\mathrm{K}} n_i (V_i - V_{\mathrm{K}}), \tag{S22}$$

$$I_{\mathrm{Ca},i} = \bar{g}_{Ca} m_{\infty,i}(V_i)(V_i - V_{\mathrm{Ca}}), \tag{S23}$$

$$I_{\mathrm{K(Ca)},i} = g_{K(Ca),i}(V_i - V_{\mathrm{K}}), \tag{S24}$$

$$I_{\mathrm{K(ATP)},i} = g_{K(ATP),i}(V_i - V_{\mathrm{K}}), \tag{S25}$$

where $V_{\mathrm{K},i}$ and $V_{\mathrm{Ca},i}$ symbolize the reversal potentials and the ion channel conductance are given by $\bar{g}_{\mathrm{K}}$, $\bar{g}_{\mathrm{Ca}}$, $g_{\mathrm{K(Ca)},i}$ and $g_{\mathrm{K(ATP)},i}$. The last two conductances are defined by the equations:

$$g_{\mathrm{K(Ca)},i} = \bar{g}_{\mathrm{K(Ca)}} \frac{Ca_{c,i}^2}{K_D^2 + Ca_{\mathrm{c},i}^2}, \tag{S26}$$

$$g_{\mathrm{K(ATP)},i} = \bar{g}_{\mathrm{K(ATP)}} o_{\infty,i}\left(ADP_{\mathrm{C},i}, ATP_{\mathrm{C},i}\right). \tag{S27}$$

The activation kinetics of the Ca^{2+} channel is given by $m_{\infty,i}$ and is defined as:

$$m_{\infty,i}(V_i) = \left(1 + e^{-\frac{-V_i+20}{12}}\right)^{-1}. \tag{S28}$$

The K^+ channels activation kinetics is governed by the variable n_i which is given by:

$$\frac{\mathrm{d}n_i}{\mathrm{d}t} = -\frac{n_\infty(V_i) - n_i}{\tau_n}, \tag{S29}$$

$$n_{\infty,i}(V_i) = \left(1 + e^{-\frac{-V_i+16}{5}}\right)^{-1}. \tag{S30}$$

The conductance of the K(ATP) channels changes rapidly in accordance to the nucleotide levels given by:

$$o_{\infty,i}\left(ADP_{\mathrm{C},i}, ATP_{\mathrm{C},i}\right) = \frac{0.08\left(1+\frac{2MgADP^{-}{}_{i}}{17\mu\mathrm{M}}\right)+0.89\left(1+\frac{2MgADP^{-}{}_{i}}{17\mu\mathrm{M}}\right)^{2}}{\left(1+\frac{2MgADP^{-}{}_{i}}{17\mu\mathrm{M}}\right)^{2}\left(1+\frac{ADP^{3-}{}_{i}}{26\mu\mathrm{M}}+\frac{ADP^{4-}{}_{i}}{1\,\mu\mathrm{M}}\right)}. \tag{S31}$$

here $ADP_{\mathrm{c},i}$ and $ATP_{\mathrm{c},i}$ are cytosolic concentrations,which in turn affect the nucleotide concentrations as desribed by:

$$2MgADP^{-}{}_{i} = 0.165APD_{\mathrm{c},i}. \tag{S32}$$

$$ADP^{3-}{}_{i} = 0.135APD_{\mathrm{c},i}, \tag{S33}$$

$$ADP^{4-}{}_{i} = 0.05ATP_{\mathrm{c},i}, \tag{S34}$$

$$\frac{\mathrm{d}ADP_{\mathrm{c},i}}{\mathrm{d}t} = J_{\mathrm{hyd,i}} - \kappa J_{\mathrm{ANT,i}}. \tag{S35}$$

The parameter κ in Eq. (S35) stands for the mitochondria/cytosol volume ratio and the terms $J_{\mathrm{hyd},i}$ and $J_{\mathrm{ANT,i}}$ (see Eq. S15) represent cytosolic hydrolysis of ATP and ATP- ADP excange. $J_{\mathrm{hyd},i}$ is modeled as a linear functions of $Ca_{\mathrm{c},i}$ as:

$$J_{\mathrm{hyd},i} = \left(k_{\mathrm{hyd}}Ca_{\mathrm{c},i} + k_{\mathrm{hyd,bas}}\right)ATP_{\mathrm{c},i}. \tag{S36}$$

In order take into accoutn other sources of hydrolysis Eq. (36) incoorporates $k_{\mathrm{hyd,bas}}$ representing a basal level of hydrolysis.

The overall concentration of cytosolic adenine nucleotide is assumed to be conserved, hence:

$$A_{\mathrm{c,tot}} = ADP_{\mathrm{c},i} + ATP_{\mathrm{c},i}. \tag{S37}$$

Cytosolic Ca^{2+} concentration is determined by the fluxes $J_{\mathrm{mem},i}$, $J_{\mathrm{er},i}$ and $J_{\mathrm{m},i}$ as it is given by differential equation:

$$\frac{\mathrm{d}Ca_{\mathrm{c},i}}{\mathrm{d}t} = f_{\mathrm{c}}\left(J_{\mathrm{mem},i} + J_{\mathrm{er},i} + \kappa J_{\mathrm{m},i}\right). \tag{S38}$$

The flux of Ca^{2+} across the plasma membrane is governed by:

$$J_{\mathrm{mem},i} = -\left(\alpha I_{\mathrm{Ca},i} + k_{\mathrm{PMCA}}\left(Ca_{\mathrm{c},i} - Ca_{\mathrm{bas}}\right)\right), \tag{S39}$$

where α is a current to flux transformation parameter, k_{PMCA} is the plasma membrane pump rate and Ca_{bas} is the bassal Ca^{2+} cytosolic concetration parameter.

The dynamics of ER Ca^{2+} concetration $J_{\mathrm{er},i}$ is governd by the the outflux modeled based on leakage $J_{\mathrm{leak},i}$ and the influx through SERCA $J_{\mathrm{SERCA},i}$:

$$J_{\mathrm{leak},i} = -p_{\mathrm{leak}}\left(Ca_{\mathrm{er},i} - Ca_{\mathrm{c},i}\right), \tag{S38}$$

$$J_{\mathrm{SERCA},i} = k_{\mathrm{SERCA}} Ca_{\mathrm{c},i}, \tag{S39}$$

$$J_{\mathrm{er},i} = J_{\mathrm{leak},i} - J_{\mathrm{SERCA},i}. \tag{S40}$$

The Ca^{2+} concentration in the ER can then bi computed as:

$$\frac{\mathrm{d}Ca_{\mathrm{er},i}}{\mathrm{d}t} = f_{\mathrm{er}}\left(\frac{V_{\mathrm{c}}}{V_{\mathrm{er}}}\right) J_{\mathrm{er},i}, \tag{S41}$$

where f_{er} is the fraction of free Ca^{2+} in the ER, and $V_{\mathrm{c},i}$, $V_{\mathrm{er},i}$ are the volumes of the cytosolic and ER compartments, respectively. Values for the parameters of the electrical/calcium model are given in Table 3. Note that for the parameters $\overline{k_{\mathrm{GPDH},i}}$, $\overline{V_{\mathrm{max},i}}$, $\overline{J_{\mathrm{GPDHbas},i}}$, $\overline{C}$, $\overline{\bar{g}_{\mathrm{K(ATP)},i}}$ the average values are given, since a dispersion of these parameters is introduced in the calculations in order to account for beta cell heterogeneity (see Materials and Methods section).

Table 3: Parameter values for the electrical/calcium compartment.

$\bar{C}_i = 5300$ pF	$\tau_{\mathrm{n}} = 20$ ms	$\bar{g}_k = 2700$ pS	$\bar{g}_{\mathrm{Ca}} = 1000$ pS
$\bar{g}_{\mathrm{K(Ca)},i} = 300$ pS	$\overline{\bar{g}_{\mathrm{K(ATP)},i}} = 15000$ pS	$V_{\mathrm{K}} = -75$ mV	$V_{\mathrm{Ca}} = 25$ mV
$K_{\mathrm{D}} = 0.5$ μM	$k_{\mathrm{hyd}} = 5\ 10^{-5}$ ms^{-1}μM^{-1}	$k_{\mathrm{hyd,bas}} = 5 \times 10^{-5}$ ms^{-1}	$\kappa = 0.07$
$A_{\mathrm{c,tot}} = 190$ mV	$\mathrm{AMP_c} = 500$ μM	$\alpha = 4.5 \times 10^{-6}$ μM ms^{-1}	$k_{\mathrm{pmca}} = 0.1$ ms^{-1}
$p_{\mathrm{leak}} = 2 \times 10^{-4}$ ms^{-1}	$k_{\mathrm{SERCA}} = 0.4$ ms^{-1}	$f_{\mathrm{c}} = f_{\mathrm{er}} = 0.01$	$V_{\mathrm{c}}/V_{\mathrm{er}} = 31$
$Ca_{\mathrm{bas}} = 0.05$ μM			

Supplementary References

[1] Bertram R, Satin LS, Pedersen MG, Luciani DS, Sherman A. »Interaction of glycolysis and mitochondrial respiration in metabolic oscillations of pancreatic islets«. Biophys J 92: 1544–1555, 2007.

[2] Smolen, P. 1995. A model for glycolytic oscillations based on skeletal muscle phosphofructokinase kinetics. J. Theor. Biol. 174:137–148.

[3] Matschinsky FM. »A lesson in metabolic regulation inspired by the glucokinase glucose sensor paradigm«. Diabetes, 45 (1996) 223-241.

[4] Bertram, R., M. G. Pedersen, D. S. Luciani, and A. Sherman. 2006. A simplified model for mitochondrial ATP production. J. Theor. Biol. 243:575–586.

[5] Bertram, R., and A. Sherman. 2004. A calcium-based phantom bursting model for pancreatic islets. Bull. Math. Biol. 66:1313–1344.

2 Supplementary Figures

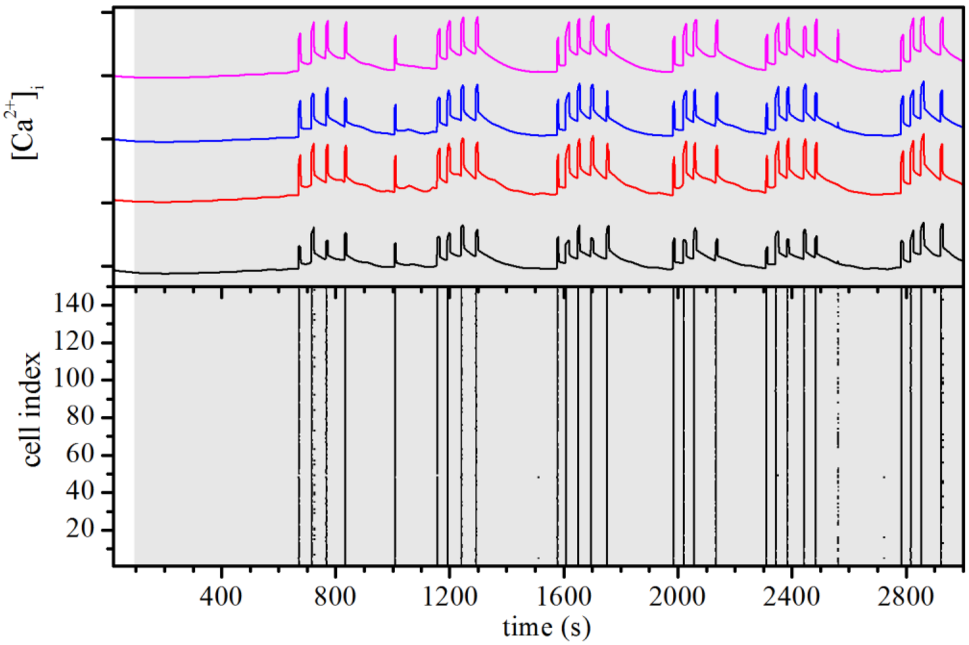

Supplementary Figure 1. Simulated spatio-temporal activity under constant stimulation with homogeneous coupling. Typical computed $[Ca^{2+}]_c$ responses of four different beta cells after switching to stimulatory conditions (upper panel, grey area indicates stimulatory conditions) and binarization of the computed oscillations of all cells (lower panel). The electrical coupling coefficient was distributed normally with mean 200 pS and relative SD of 30 %. In this case very synchronized behavior is obtained without progressive and heterogeneous activations of cells, as observed in experiments.

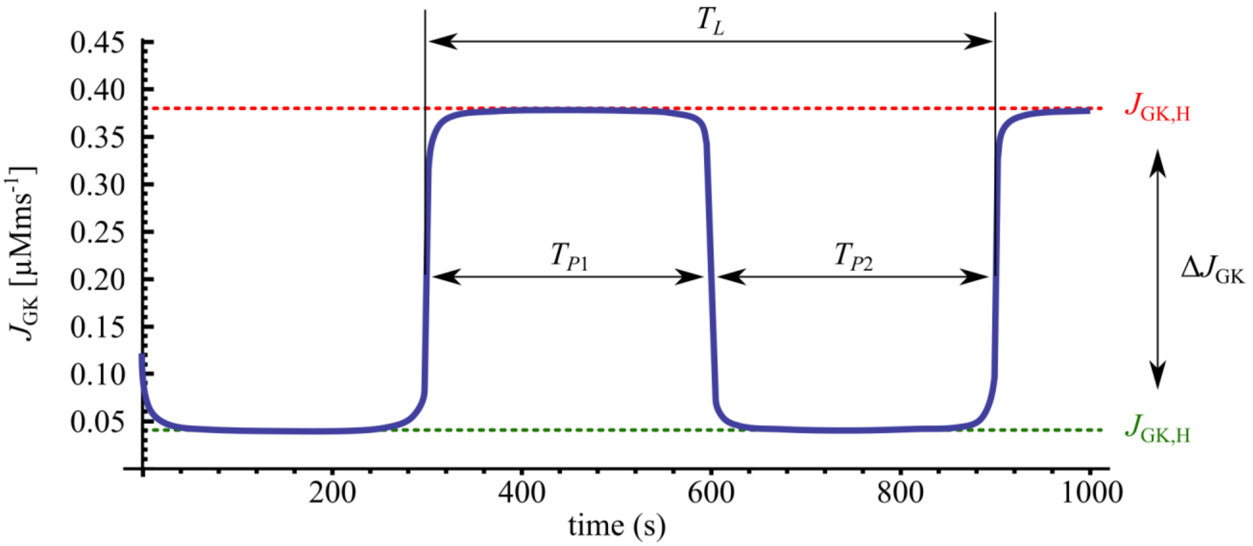

Supplementary Figure 2. The course of the simulated oscillatory stimulation protocol. Variations in the glucokinase reaction rate reflect the oscillatory changes in glucose.

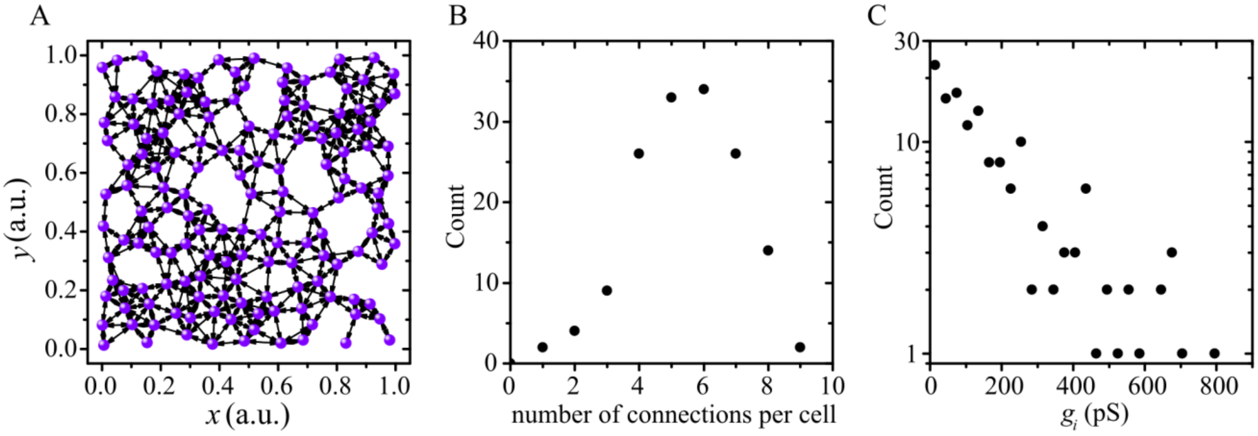

Supplementary Figure 3. Coupling in the multicellular beta cell model. A) A typical structure of the intercellular network of beta cells. B) The corresponding degree distribution. The beta cell network is quite homogeneous with a mean degree around 6. C) The distribution of the electrical coupling coefficient. The coupling strength is rather heterogeneous and follows an exponential distribution with a mean of 200 pS.
